# A conserved minimal core and modular extensions make the fungal flagellum

**DOI:** 10.1038/s41598-026-52644-y

**Published:** 2026-05-12

**Authors:** Aleksander Kossakowski, Drishtee Barua, Anna Muszewska

**Affiliations:** 1 Institute of Biochemistry and Biophysics, Polish Academy of Sciences, Pawińskiego 5A, 02-106 Warsaw, Poland; 2 Doctoral School of Molecular Biology and Biological Chemistry at IBB PAS, Pawinskiego 5a, 02-106 Warsaw, Poland

**Keywords:** Fungi, Motility, Flagellum, Cell biology, Microbiology

## Abstract

Flagellated fungi are pivotal for understanding the evolution of eukaryotic motility, yet knowledge of their flagellar proteins remains fragmentary. We assembled a cross-phyla atlas by mapping 394 reference flagellar proteins on 184 fungal proteomes. Out of 394 orthogroups, 342 orthogroups have fungal homologs, of which 210 orthogroups are specific to flagellated fungi. From these, we propose a 184-orthogroup minimal flagellum encompassing proteins shared by flagellated fungi. This minimal toolkit retains the axonemal core (tubulins, dynein arms), beat regulators (nexin–dynein regulatory complex, radial spokes), intraflagellar transport (IFT-B, IFT-A, IFT kinesin, dynein), basal-body and trafficking (exocyst/TRAPP). Genes specific to the flagellum preferentially express during flagellated life cycle stages; but conserved flagellar genes show no such expression specificity, regardless of whether the species is flagellated. Fungal lineages differ in the composition of flagellar modules. Neocallimastigomycota stand out as the most diverged among flagellated lineages with the broadest flagellar toolkit, including a complete BBSome, yet reduced centriole-associated modules. We delineate a conserved engine for fungal motility and a modular, lineage-specific set of proteins that broadens regulation and sensory capacity. The conservation of flagellar proteins between fungi and animals opens up possibilities of experimental functional validation in models simpler than animals.

**Supplementary Information:**

The online version contains supplementary material available at 10.1038/s41598-026-52644-y.

## Introduction

Fungi are one of the most diverse and complex kingdoms on Earth, with an estimated number of species reaching up to thirteen million^1^. These organisms occupy a wide range of ecological niches, including soil, water, air and form a plethora of relationships with other living forms^2^. Within the multiplex fungal tree of life, there are two main fungal supergroups - Dikarya and non-Dikarya, which encompass many fungal phyla - from symbiotic Glomeromycota to obligatory parasitic Microsporidia^3^. Some non-Dikarya fungi have a single or multiple posterior flagella on their zoospores. They are flagellated fungi, which currently consists of: Blastocladiomycota, Chytridiomycota (chytrids), Neocallimastigomycota, Olpidiomycota, Monoblepharidiomycota *and Rozella allomycis* (Rozellomycota)^4^. Chytrids have a dimorphic life cycle, with a motile free-swimming uniflagellate zoospore and a sessile walled thallus with rhizoids^5^. The single posterior flagellum can round the zoospore twice^5^ and upon transition to the immotile thallus it is degraded by the proteasome^6^.

A complex motility machinery allowed flagellated fungi to colonize substrates and grow in aquatic environments^7,8^. The function of appendices such as flagellum required the orchestration of the cytoskeletal components^9^. It is currently acknowledged that flagellated fungi harbor cytoskeletal components related to their animal counterparts^10^.

Many motility-related genes were lost in Dikarya but retained in a majority of the remaining Opisthokonta lineages^2,11^. The investigation of flagellar proteins has been primarily focused on animal systems. Consequently, fungal phylogenomic studies often characterize flagellar proteins by reference to well-studied animal homologs, including mouse, rat, fruit fly, and human. These organisms are commonly used as models for human infertility^12,13^. The limited research on fungal flagella highlights the need for comprehensive studies, built on well-characterized animal references.

Flagellar movement in fungal zoospores is driven by the microtubule based axoneme and associated motors, but flagellated fungi also rely on a broad cytoskeletal toolkit that includes actin regulators characterized in metazoans. For instance, Prostak et al. (2021)^14^ showed that *Batrachochytrium dendrobatidis* zoospores build dynamic, animal-like actin networks and encode actin regulators and myosin motors that are shared between chytrids and animals, supporting actin dependent behaviors that accompany the motile stage, rather than powering flagellar beating itself^14^. Additionally, comparative analyses have shown that Chytridiomycota retains genes associated with centriole duplication, ciliary membrane docking, and basal body organization^13^. This highlights the conservation of key centrosomal and basal body components in chytrids, many of which are shared with metazoans.

At the core of the canonical flagellum are microtubules, which are tubular structures composed of subunits known as tubulins. Microtubules form the axoneme of flagellum, providing structural support and stability. Diversity of eukaryotic cilia and flagella is commonly described in terms of axonemal architecture and lineage-specific accessory modules^15^. Canonically, flagella exhibits a “9 + 2” axoneme, where nine outer microtubule doublets surround a central pair, exemplified by the biflagellate green algae *Chlamydomonas reinhardtii*^16^. Many sensory primary cilia lack the central pair (and are referred to as “9 + 0”) however importantly, “9 + 0” axonemes can also be motile in specific contexts, such as vertebrate nodal cilia^17^. Comparative studies further show that a conserved basal body and axonemal core coexists with lineage-specificity that tune hydrodynamics and regulation, including tripartite mastigonemes on the anterior flagellum of heterokonts (for example in stramenopiles and oomycete zoospores)^18,19^ and extra-axonemal scaffolds such as the paraflagellar rod in kinetoplastids (for example *Trypanosoma brucei*)^20^. In this framework, flagellated fungi represent the opisthokont configuration, typically producing a smooth posterior whiplash flagellum on zoospores^7^.

Motor proteins, particularly axonemal dyneins and kinesins, are essential for flagellar motility. Axonemal dyneins generate force by ATP driven sliding between adjacent microtubule doublets, which is converted into bending and produces the characteristic beat^21^. Disruption of core dynein components gives clear functional phenotypes: pathogenic variants in outer dynein arm genes frequently lead to primary ciliary dyskinesia or multiple morphological abnormalities of the flagella (MMAF) with absent or reduced outer dynein arms, reduced beat frequency, and clinical features such as chronic pulmonary disease and male infertility due to immotile sperm flagella^22^. MMAF cases seem to be related to diverse mutations in genes encoding dyneins (light chains: DNALI1^23^, or heavy: DNAH1^24^, DNAH2^25^, DNAH6^26^, DNAH7^27^, DNAH8^28^, DNAH10^25^,DNAH12^29^,DNAH17^30^) and cilia- and flagella-associated proteins CFAP47^31^, CFAP44^32^, CFAP45^32^, CFAP57^33^ and CFAP65^34^ CFAP69^35,36^, CFAP70^37^, CFAP251^38,39^ influencing indirectly or directly the central pair of axonemal microtubules of the sperm flagella^24^. Kinesins primarily support intraflagellar transport (IFT) and the delivery of axonemal and membrane proteins required for assembly and maintenance of flagella^21^; disease causing variants in kinesin 2 subunits (for example KIF3B) impair anterograde IFT and are associated with ciliopathy phenotypes^21,40,41^. The intraflagellar transport is based on two IFT complexes referred to as IFT-A and IFT-B. BBSome complex works together with the IFT and membrane proteins ensuring proper compartmentalization of signaling molecules^42^. Malfunction of the BBSome results in mislocalization of membrane receptors, disturbed signal transduction, and in consequence ciliopathies^43,44^. Radial spokes are multiprotein complexes connecting the outer doublets to the central pair and contribute to dynein regulation. Consistent with this regulatory role, variants in radial spoke head genes (for example RSPH4A) disrupt axonemal ultrastructure and are linked to abnormal beating patterns^45^. Nexin linkers, now considered part of the nexin dynein regulatory complex (N-DRC), mechanically couple neighboring doublets, limit excessive sliding, and coordinate dynein activity across the axoneme; structural and genetic studies support that defects in N-DRC components (for example DRC1) can produce motility failures and primary ciliary dyskinesia related phenotypes^46,47^. In addition to these core structural modules, many other proteins contribute to motility related processes including sensory signaling, membrane biogenesis, and control of flagellar length and waveform. For example, mutations in INPP5E, a ciliary phosphoinositide 5 phosphatase required for proper ciliary signaling, are associated with ciliopathy phenotypes^48–50^.

Here we present an expanded and refined flagellar protein atlas across fungi. We denote differences between flagellated fungal lineages and conservation of some of the flagellar structures regardless of taxonomic positioning.

## Results

### Dataset construction and orthogroup identification

To identify the set of flagellar proteins in fungi, we scanned 184 fungal proteomes from all fungal phyla for the presence of flagellar proteins from a reference dataset (Supplementary File S1). We created this reference set of 394 flagellar proteins by combining data from Complex Portal^51^(*n* = 188), CORUM^52^(*n* = 210), UniProtKB^53^(*n* = 261), as well as from the works of Merenyi et al. 2023^11^, (*n* = 87), Pazour et al. 2005^54^, (*n* = 123) and Long et al. 2025^13^, (*n* = 62). These reference proteins include mainly human and *Chlamydomonas reinhardtii* homologs. We collected the orthologs of these 394 proteins creating 394 orthogroups, which allowed us to gather homologs bridging the animal and fungal evolutionary gap. We performed phylogenetic analyses for orthogroups with numerous paralogs, such as calmodulin (CALM1/2/3), dynein heavy chains (such as DNAH9/11/17) or cytoplasmic dynein light chains (DYNLL1/2, Supplementary Figures S3 - S14). Mapping of the reference flagellar orthogroups on fungal proteomes showed no homologs for 52 orthogroups, suggesting their absence in fungi. From those 52 orthogroups, four are retained in *Chlamydomonas reinhardtii*, in addition to other opisthokonts, for example Tektin and its paralogs (Supplementary File S1). Those missing homologs in fungi could be explained by evolutionary distance between algae and fungi. Most of the orthogroups absent from fungi (30/52) have a disordered region equal or greater than 30% of the protein sequence. Many of these proteins have Metazoa specific domains, for instance SPATA7 with HSD3 domain (PF15244) or KIAA0753 with ‘protein moonraker’ (PF15718) domain. On the other hand, 14/52 orthogroups do not have a Pfam domain (Supplementary File S1). The limited taxonomic distribution of aforementioned proteins and their domains suggests that at least some of the orthogroups without fungal homologs could be animal specific (instead of being lost in fungi). In addition, the abundance of disordered regions complicates the process of finding homologs.

**Fig. 1:**
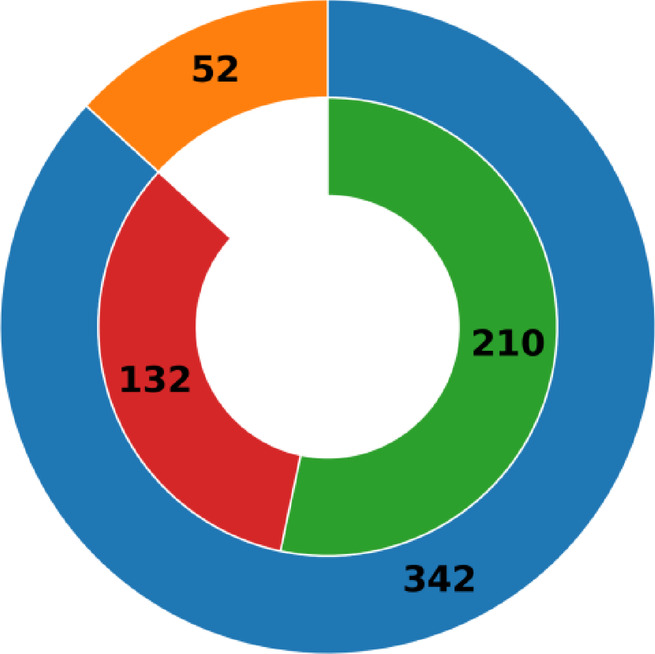
Distribution of 394 flagellar orthogroups across 184 fungi. Orange - orthogroups without fungal homologs, blue - orthogroups with fungal homologs, red - orthogroups occurring both in flagellated and non-flagellated fungi, green - orthogroups specific to flagellated fungi.

From the remaining 342 orthogroups which have fungal homologs, less than half (*n* = 132) occur also in non-flagellated fungi, while the majority of orthogroups (*n* = 210) occur only in flagellated fungal phyla (Blastocladiomycota, Chytridiomycota, Monoblepharidomycota, Neocallimastigomycota, Olpidiomycota, Rozellomycota, Fig. 1). We define the 210 flagellated-specific orthogroups as occurring in flagellated fungal species within the aforementioned phyla, but with a possible one residual protein (or incomplete loss) within non-flagellated organisms.

Flagellated fungi specific orthogroups have more transmembrane helices, intrinsic disorder regions and repeat-type scaffolding domains, most notably WD40 (PF00400, PF15911), LRR (PF13516, PF14580), Armadillo (PF00514) and TPR (CL0020) repeats (Table 1). Collectively, these features are consistent with the assembly of flagellar structures, including axonemal subcomplexes, motor associated elements and associated signalling modules. By contrast, orthogroups that are not specific to flagellated fungi are enriched in signal peptides and catalytic and motor domains, such as Pkinases (CL0016, Table 1, Supplementary File S1). These proteins contribute to broadly conserved roles in cytoskeletal dynamics, signalling and intracellular transport that are shared across fungi regardless of whether a flagellum is retained. Taken together, the flagellated specific set consists of proteins that build and regulate the flagellum, whereas the non-specific set largely reflects conserved cellular functions that are widespread across the fungal kingdom.

**Table 1:** Feature comparison between 210 flagellated-fungi specific orthogroups and 132 orthogroups shared between fungi with and without flagellum.

Orthogroups	Specific to flagellated fungi	Non-specific to flagellated fungi
Number of fungi	21	184
Number of orthogroups	210	132
Total fungal proteins	4 405	33 061
Orthogroups in which every protein contains ≥ 1 coiled-coil	95 / 210	33 / 132
Orthogroups in which every protein contains ≥ 1 low-complexity region	177 / 210	110 / 132
Orthogroups with proteins containing a signal peptide	23 / 210	73 / 132
Average fraction of intrinsic disorder per orthogroup	23%	21%
Orthogroups in which every protein contains ≥ 1 transmembrane region	16 / 210	7 / 132
Most common protein domains	35 / 210 orthogroups carry WD40, LRR, Armadillo or TPR repeats	12 / 132 orthogroups carry Pkinase domain

**Fig. 2:**
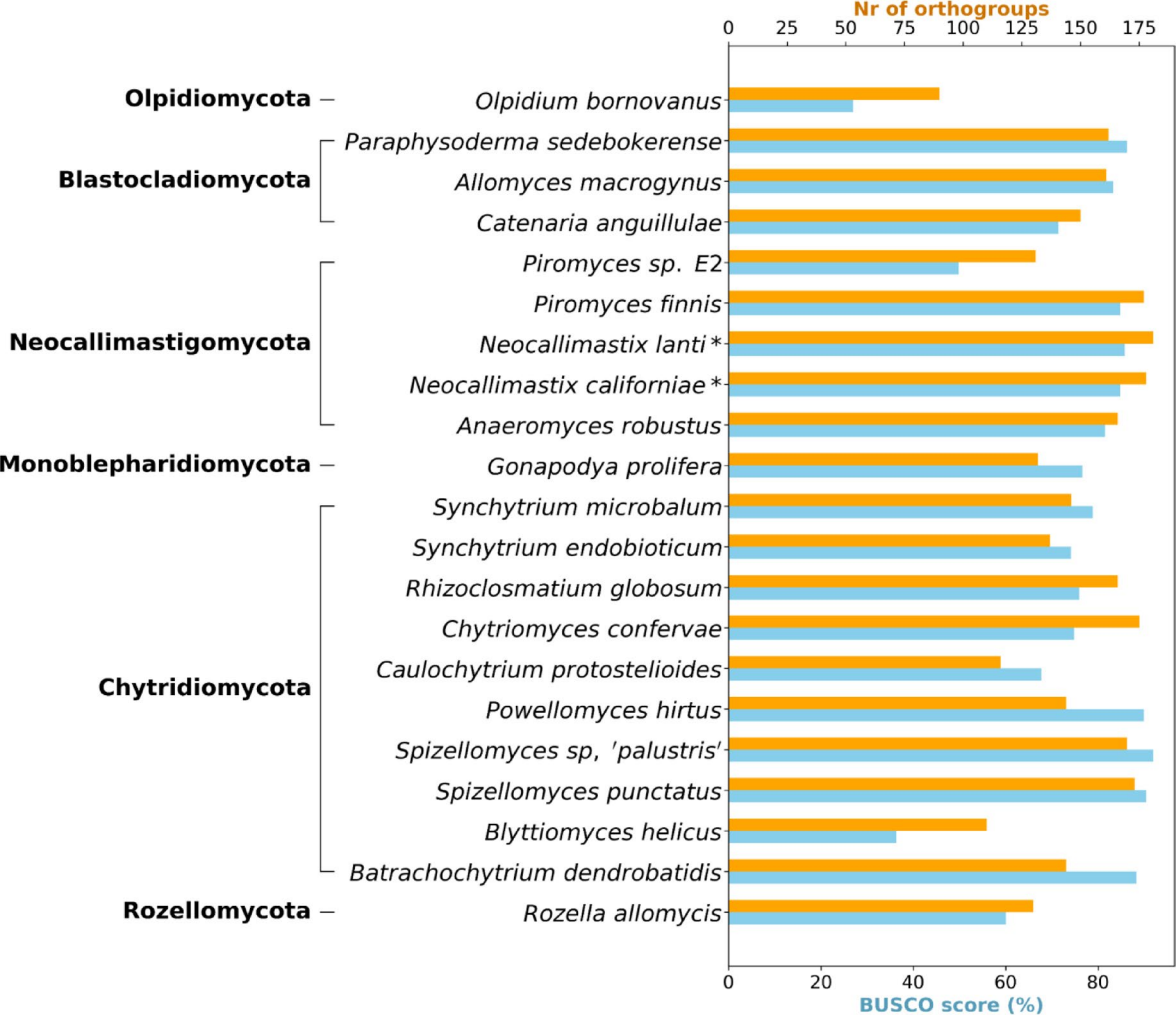
Per-species retention of flagellated-fungi–specific orthogroups, that include at least one protein from each flagellated fungal species (orange), with BUSCO score for each specimen (blue). Taxonomic nomenclature based on Wijayawardene et al. (2024)^4^. Species denoted with an asterisk (*) can have multiple flagella in their zoospores.

The number of flagellum-specific orthogroups retained in individual fungal isolates varies, however all three top scoring organisms in terms of number of orthogroups retained belong to Neocallimastigomycota. Out of 210 orthogroups present only in flagellated organisms, *Neocallimastix lanti* (BUSCO score 85.8) retained 182, while *Neocallimastix californiae* (BUSCO score 84.7) have retained 179. On the other extreme, *Olpidium bornovanus* retained 91 of 210 orthogroups. However, it should be noted that the BUSCO score for this species genome is low (27) and the relatively low number of flagellum-specific orthogroups could also be explained by the species parasitic genome reduction. The next species with the lowest number of flagellated fungi-specific orthogroups is *Blyttiomyces helicus* (BUSCO score 36,3) with 111 of 210 orthogroups retained (Fig. 2, Supplementary File S1, Supplementary Figure S1).

Taken together, the patterns of protein retention point to the existence of a subset of core proteins which are maintained across flagellated fungi. Identifying these conserved components can provide insights into the minimal requirements for constructing and operating a functional fungal flagellum.

### Minimal flagellum proteome

From the 342 flagellar orthogroups identified in fungi, 184 occur in at least 18 flagellated fungal species ( > = 85% of taxa), which has been taken as a minimal criterion to consider a given orthogroup as constituting a potential minimal flagellum (Supplementary File S1). A minimal flagellum is the smallest, self-sufficient set of flagellar modules that still supports axoneme assembly and rhythmic beating.

In our dataset, the minimal toolkit retains a “9 + 2” axoneme with canonical tubulins, central pair and radial spoke components, and both outer and inner dynein arm modules with key regulators. Assembly and upkeep are supported by a core IFT system (primarily IFT-B with a minimal IFT-A subset), the IFT motors, and a flagellar tip kinesin. At the base, the set includes centriole duplication and stability factors, a defined transition zone cohort, septins, vesicle delivery machinery (exocyst and TRAPP), and a small set of flagellar membrane proteins (Table 2, Supplementary Table S1).

Compared with the full flagellar set of proteins, the “minimal flagellum” lacks many accessory systems implicated in gating, cargo selection, and signalling, including the IFT-BBSome core and CatSper-type channels. It also lacks numerous distal appendage, pericentriolar, and transition zone add-ons, as well as expanded centriole-associated and auxiliary motility and trafficking factors (Supplementary File S1), consistent with a streamlined but functional flagellar proteome.

There are 103 flagellated-fungi specific groups in the minimal flagellar set, out of 210 total flagellated-fungi specific orthogroups.

**Table 2:** Comparison of flagellated-fungi specific orthogroups occurring in minimal flagellum and orthogroups in non-minimal set.

	Minimal flagellum (184/342)	Non-minimal flagellum (158/342)
Number of flagellated fungi specific orthogroups	103	107
Total fungal proteins	2 958	1 447
Orthogroups in which every protein contains ≥ 1 coiled-coil	56 / 103	38 / 107
Orthogroups in which every protein contains ≥ 1 low-complexity region (LCR)	91 / 103	86 / 107
Orthogroups in which every protein contains ≥ 1 trans-membrane helix	–	16 / 107
Domain enrichment in proteins	25 / 103 orthogroups with WD40, LRR, TPR or Armadillo repeats	4 / 107 orthogroups with Ion_trans, 4 / 107 with 7tm_1 domains and 10 / 107 non-domain

**Fig. 3:**
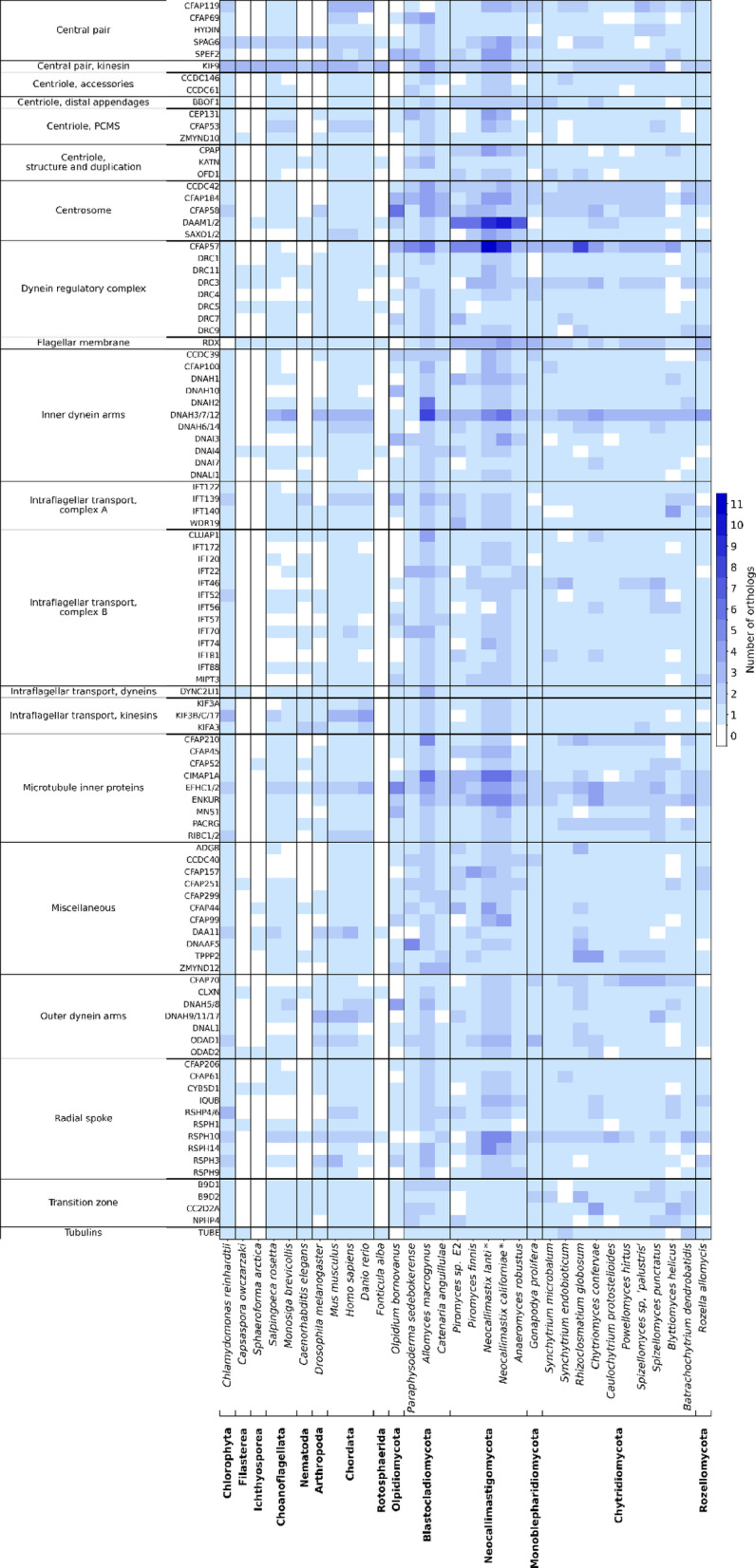
Taxonomic distribution of 103 flagellated-fungi specific orthogroups within the minimal flagellum set, categorized into complexes based on the works of Merényi et al. (2023)^11^ and Long et al. (2025)^13^ as well as CORUM, ComplexPortal and other sources (Supplementary Table S1); Centriole, PCMS - Pericentriolar Material and Satellites. Taxonomic order based on Wijayawardene et al. (2024)^4^ and Liu et al. (2024)^55^. Species denoted with * can have multiple flagella in their zoospores.

The 103 minimal-flagellum orthogroups specific to flagellated fungi are strongly biased toward coiled-coil regions, low-complexity regions and WD40 (PF00400, PF15911), LRR (PF13516, PF14580), Armadillo (PF00514) and TPR (CL0020) scaffolding repeats, while being depleted in transmembrane helices. This points to flexible adapters that link and stabilize the axoneme and its motors without relying on membrane trafficking (Table 2; Fig. 3, Supplementary File S1).

In addition, we observe a significant increase in the number of copies of DAAM1/2 and CFAP57 homologs in Neocallimastigomycota, as well as significant increase of CFAP57 homologs in *Rhizoclosmatium globosum*. In our data, we can observe that Neocallimastigomycota have slightly higher copy numbers of homologs than other phyla. This is especially visible for DAAM1/2 and CFAP57, with the number of copies, ranging from six up to ten copies per organism **(**Fig. 3, Supplementary Figure S2). Dishevelled-associated activator of morphogenesis 1 and 2 (DAAM1/2) bind regulatory GTPases and contain TPR domain. DAAM1/2 promotes actin filament nucleation and elongation, contributes to ciliogenesis, and is required for myocardial maturation and sarcomere assembly. It may also regulate RHOA activation, thereby influencing mitotic spindle orientation and chromosome segregation 56. Cilia- and flagella-associated protein 57 (CFAP57) is associated with dynein regulatory complex and thought to be associated with inner dynein arms. This protein is composed of WD40 repeats 57 and might be a scaffolding structural element.

The 107 non-minimal flagellated-fungi specific orthogroups are enriched in transmembrane helices, which are present in Ion_trans (PF00520) and 7tm_1 (PF00001) domains, in addition to ten orthogroups without any Pfam domains (Table 2). Numerous transmembrane domains are needed for flagellar membrane proteins, including G protein-coupled receptors such as DRD2 or CCR6 as well as CatSper channel subunits such as CATSPER1 or CATSPER2. Both CatSper channel complex and BBSome complex components are not universally retained across flagellated fungi suggesting a differentiation in the interflagellar transport among these organisms (Supplementary File S1).

**Fig. 4:**
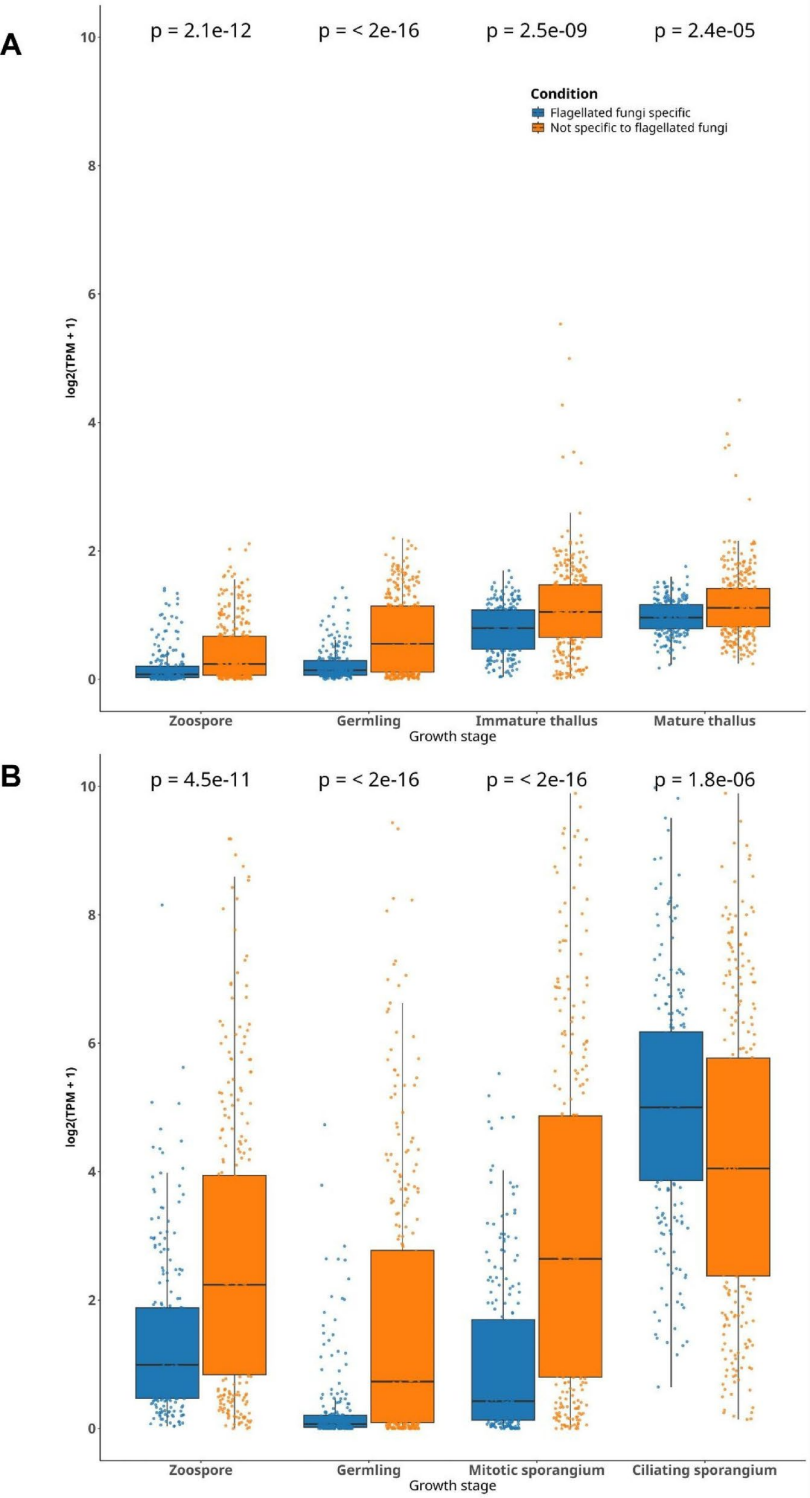
Changes in expression levels of flagellar genes across life stages of *Rhizoclosmatium globosum.* Boxplots show log₂-transformed average Transcripts Per Million (TPM) values of flagellar genes belonging to orthogroups specific to flagellated fungi (in blue) and present across fungi regardless of the presence of flagellum (in orange). (**A**) dataset A in four growth phases: zoospore, germling, immature and mature thallus, as defined by Laundon et al., 2022^5^; (**B**) dataset B in four growth phases: zoospore, germling, mitotic and ciliating sporangium, as defined by Chrismas et al., 2025^58^. Mann-Whitney U Test* p*-values are shown for the comparison of expression levels between flagellum specific and non-specific genes within each life stage.

In order to determine the time and level of expression of flagellated-fungi specific and flagellated non-specific genes, we re-analysed datasets originating from two experiments on the life cycle of the chytrid *Rhizoclosmatium globosum* (Supplementary File S1). The datasets are referred to as dataset A^5^ and dataset B^58^. Out of the 547 flagellar protein encoding genes in *R. globosum* identified in our study, 512 were expressed in dataset A (Fig. 4A) and 525 in dataset B (Fig. 4B).

In general, genes belonging to orthogroups specific to flagellated fungi show lower expression levels than genes belonging to orthogroups conserved across fungi (in both datasets), which has been confirmed by Mann-Whitney U Test (Fig. 4). In addition, the expression values of flagellated-fungi-specific orthogroups are more tightly distributed around the median, whereas orthogroups conserved across fungi display greater dispersion and a higher number of outliers. The main exception is the ciliating sporangium growth phase (Fig. 4B), in which flagellated-fungi-specific orthogroups show a higher median expression level. These results point at a regulated expression of flagellar genes during the fungal life cycle.

### Phylum based results

**Fig. 5:**
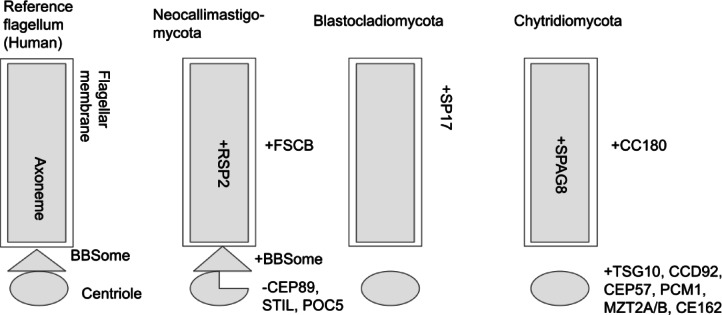
Differences in the conservation of flagellar modules between major flagellated fungal phyla (Blastocladiomycota, Neocallimastigomycota, Chytridiomycota). Presence is denoted by “+”, absence by “-”.

While a conserved core of flagellar proteins can be defined across fungi, closer inspection reveals clear phylum-level differences in the retention and composition of individual complexes (Fig. 5). One such example can be the BBSome, which is universally retained in Neocallimastigomycota (P. *finnis*,* Piromyces sp. E2*,* A. robustus*,* N. lanti* and *N. californiae*), encoding all eight components. In contrast, Chytridiomycota exhibit a fragmented BBSome core, with only a subset of subunits present, most of which observed in *C. confervae* which retains five subunits (BBS1, BBS4, BBS5, BBS7, TTC8), *R. globosum* and *B. helicus* retain four, *C. protostelioides* - one, while the remaining chytrids lack the complex entirely. No BBSome-specific proteins are found in the other fungal phyla proteomes used in this study.

Aside from BBsome being a major differentiator between Neocallimastigomycota and the remaining fungal phyla, we can observe phyla-specific differences within orthogroups. Although there are no more phyla-specific complexes other than BBSome that are conserved entirely in one phylum, we observe 36 orthogroups with differing phyla protein distributions (Fig. 5).

Neocallimastigomycota lacks 20 out of 36 fungal orthogroups, of which nine are associated with centrioles and three out of nine are present in at least one other fungal phyla. In addition, there are eight groups occurring only in Chytridiomycota and one that occurs only in Blastocladiomycota.

## Discussion

We performed a comprehensive search for flagellar proteins across diverse fungal representatives which enabled us to explore the evolutionary trajectories of flagellum building blocks. Our study expanded previous studies considering either a narrower set of flagellar proteins^11^, a well defined flagella-related structure such as centriole^13^ or featured diverse organisms with only a few flagellated fungi^59^.

We identified a clear split between flagellated-fungi specific orthogroups (*n* = 210) and those shared with non-flagellates (*n* = 132) which mirrors the pruning of the ancestral opisthokont ciliary toolkit. Accordingly, gene families tied to axonemal assembly, IFT scaffolding, and membrane docking (enrichment in coiled-coils, low-complexity/disorder, and repeat scaffolds such as WD40/LRR/ARM) are preferentially retained in flagellated lineages, whereas broadly useful modules (kinases, tubulins) persist across fungi regardless of flagellation. This pattern is consistent with the “delayed loss of ancestral gene families” model inferred from comparative genomics across some of the non-Dikarya^11,60^, where traits are shed piecemental and lineage-specifically rather than in a single step^11^. Conversely, enrichment in kinases/tubulins shared between flagellated and non-flagellated fungi is consistent with the description of flagella evolution in literature^11^. Moreover, it opens up the possibility of moonlighting, non-flagellar roles for many signalling and cytoskeletal proteins^54^. This is also consistent with the difference in expression profiles between these two sets of genes. We found out that the flagellum specific genes are clearly regulated during the fungal life cycle with an increased expression during ciliogenesis whereas, the flagellum-related genes present across fungi have a more unified expression profile across the life stages of *Rhizoclosmatium globosum*.

Our results point at the existence of two subsets of the fungal flagellar proteome: a conserved minimal flagellar proteome constituting the “engine” required for axoneme assembly and beating, and a more lineage-specific set of proteins that expands membrane trafficking and signalling capacity. The existence of such a conserved core set of flagellar proteins, present across fungi and other Eukaryotes is consistent with the consensus, that the last eukaryotic common ancestor already possessed a flagellum^59,61^. This ancestral flagellum likely consisted of a canonical basal body and a motile “9 + 2” axoneme maintained by IFT, with subsequent evolution dominated by differential retention and expansion of accessory structures^59^. In this context, flagellated fungi exemplify a reduction of the flagellar proteomes. In humans, the flagellum of spermatozoa contains a “9 + 2” axoneme together with peri axonemal structures, including outer dense fibers, the fibrous sheath, and a mitochondrial sheath in the midpiece^62^. Human flagellum includes many factors for dynein regulation, signaling, and local ATP production that modulate beat parameters and support motility^62,63^. Mutations in non axonemal components are associated with sperm motility defects and with broader motile cilia disorders^54,62,63^.

In addition, in humans and mice identical calmodulin proteins are encoded by three genes - CALM1, CALM2, CALM3^64^. Fungi possess either one canonical calmodulin per genome (*S. cerevisiae*^65^, *A. nidulans*^66^) or multiple calmodulins like *Rhizopus delemar*^67^. We observed that singular calmodulins are typical of Dikarya fungi, but non-Dikarya fungi can have multiple copies of calmodulins (Supplementary Figure S6).

As shown in this study, the number of orthogroups retained in individual isolates varies, however all three top scoring organisms in terms of number of orthogroups retained belong to Neocallimastigomycota. This could result from many reasons, one of the most probable would be multiflagellated zoospores of some of those fungi. While generally, flagellated fungi possess a single posterior flagellum, *Neocallimastix spp.* produces polyflagellate zoospores with a brief motile window before rapid encystment in the viscous rumen, which could lead to a more conserved and robust flagellar toolkit needed for their habitation of the demanding, dense rumen environment^68^.

Upon a more detailed inspection, Neocallimastigomycota harbour the broadest and most intact flagellar toolkit, most notably a universally retained BBSome, which ensures proper compartmentalization of signaling molecules to the axoneme, and is cooperating with IFT^42,43,69^. On the other hand Neocallimastigomycota have documented losses of centriolar protein coding genes including CEP89, STIL, POC5 in centriole-associated protein set^70^.

While currently, there are no experimental studies explaining the specific ecological role of the BBSome in fungi (Neocallimastigomycota specifically), Rossier et al. (2023) show retention of two BBSome elements in *Piromyces spp*. and loss in *A. macrogynus*,* B. dendrobatidi*s and *R. allomycis*, which aligns with our results^71^. Concurrently, despite broad axonemal/IFT retention, Neocallimastigomycota in our dataset show a marked reduction of centriole-associated modules, particularly pericentriolar material and satellites. These modules are known to fine-tune basal-body maturation, protein targeting and ciliary gating rather than build the axoneme itself^72^. This aligns with works published by Heath and co-workers describing the dismantling of the basal body when the zoospores encyst and that only zoospores have centrioles in Neocallimastigomycota^70,73^. Moreover, we observe a high copy number of DAAM1/2 and CFAP57 orthologs in Neocallimastigomycota. This could be explained by unusually large, AT-rich, repeat-laden Neocallimastigomycota genomes with extensive gene duplication and horizontal gene transfer^74^. However, the more likely scenario is vertical descendance followed by multiple duplications. While best documented for CAZymes, the duplications can be observed for other families, including flagellar modules^74^. Both DAAM1/2 and CFAP57 are repeat-rich proteins functioning in protein complexes. Their specific role in Neocallimastigomycota remains unclear. In general, paralog repertoire provides a plausible substrate for subfunctionalization, neofunctionalization, or dosage effects for interacting partners. In *Homo sapiens*, DAAM1/2 functions are not exclusively linked to filament formation^56^. One possible scenario is that Neocallimastigomycota DAAM1/2 paralogs mediate a particularly refined regulation of actin filament formation. Alternatively, some paralogs may have undergone neofunctionalization, acquiring distinct regulatory functions in other cellular compartments. CFAP57 in animals and green algae, is implicated in the asymmetric targeting and stabilization of a subset of inner dynein arms and thereby influences waveform and beating^57^. In Neocallimastigomycota, additional CFAP57 copies might contribute to the diversified dynein arm scaffolds. In *Rhizoclosmatium globosum*, the elevated copy number of CFAP57 adds to known niche-associated duplications of genes^13,58,75^. Multiple CFAP57 paralogs in *R. globosum* could therefore support fine-tuning of dynein-arms, potentially optimizing zoospore propulsion or steering. Differences in the flagellar proteome among Chytridiomycota may also be linked to the ability of some taxa to move by amoeba-like crawling in addition to flagellar propulsion, such as in *Spizellomyces punctatus*^6^.

Zoospores differ in the way they explore the environment either as a random-walk (e.g. *Allomyces macrogynus* and *Spizellomyces punctatus*) or via a circular swimming style (e.g. *Chytriomyces confervae* and *Rhizoclosmatium globosum*) however the speed they display seems to be comparable^76^. According to Galindo et al. (2024)^76^ the swimming style depends on the structure of the flagellum and tubulin assembly more than taxonomic placement. In our data random walk swimmers and circular swimmers differ by a small subset of proteins, namely TBCCD1, C2CD3, TUBGCP4, SSNA1 and NEK8, all of which are present in *A. macrogynus* and *S. punctatus*, but are absent from *C. confervae* and *R. globosum*. Those proteins could be candidates for shaping non-axonemal tubulin arrays associated with random-walk swimming.

We recovered homologs of several centrosomal or centriolar proteins, including TSG10, CCDC92, CEP57, PCM1, MZT2, and CEP162, exclusively from Chytridiomycota, with no detectable homologs in other sampled fungal phyla. Noteworthy some of these proteins were not reported in Chytridiomycota previously, for instance MZT2^13^. The conservation of multiple centriolar proteins is consistent with experimental studies 13, which show that chytrids retain a centriole and flagellum toolkit that is absent from most other fungal lineages.

One of the goals of our study was to determine which proteins of the flagellum contribute to its functional core. In consequence, we defined a minimal set of flagellum proteins conserved across flagellated taxa. This enabled us to observe that the minimal flagellum relies more on long cytosolic scaffolds than on membrane complexes: vesicles can still dock and deliver building blocks to the base, IFT trains can move them along the axoneme, and dynein arms can generate beat, but membrane trafficking, ion-channel mediated signalling and regulatory plasticity are limited. In practical terms, this minimal organelle is motile and maintainable, yet optimized for core locomotion with simplified gating and reduced sensory/trafficking capacity. The minimal flagellum can function because of the components it retains – axonemal tubulins, dynein motors, radial-spoke/nexin regulators and the core IFT-A/B with IFT dynein and IFT kinesin. In contrast, most proteins lost act upstream or peripherally: the BBSome and some auxiliary IFT that mainly recycle signalling receptors and membrane proteins. For example, both *Trypanosoma brucei* and *Chlamydomonas* mutants lacking a complete BBSome retain normal beat frequency and waveform^42,44,69^. Similarly, mammals lacking CatSper channel subunits retain progressive, low-amplitude beating but cannot hyperactivate, showing that the Ca²⁺-gated CatSper channel complex is a performance enhancer rather than an architectural necessity 77. Likewise, comparative work on IFT shows that the 11-subunit core IFT-B platform is obligatory for axoneme assembly, whereas many accessory IFT-A subunits are repeatedly lost in organisms whose flagella serve mainly locomotion purpose^78^. This “fungal minimal flagellum” is consistent with the eukaryotic ciliogenesis core inferred by Dobbelaere et al., (2023)^79^, which includes basal-body, transition-zone, IFT, dynein-arm, radial-spoke, N-DRC, and central-apparatus components^79^. The minimal flagellum set of proteins can be a shortlist for experimental studies on flagellar function and the role of individual proteins in locomotion. One might start from testing the impact of known mutations identified in patients with MMAF for instance in dynein proteins which are ubiquitously preserved in fungi like dynein light chains such as DYNLT1, DYNLT4 (Supplementary Figure S12) or dynein heavy chains like DNAH3 or DNAH9 (Supplementary Figure S14), as well as CFAP44, CFAP45 or CFAP57 homologs. Moreover, the conservation of the core flagellar elements between flagellated fungi and animals puts forward the possibility of experimentation in simple models like saprotrophic Chytidiomycota.

## Conclusions

We provide a curated, cross-phyla atlas of fungal flagellar proteins that clearly separates a flagellate-specific, scaffold-rich layer from broadly conserved housekeeping modules. From this, we propose a 184-orthogroup “minimal flagellum” capturing the axonemal core, dynein regulation, and pared IFT, while a non-minimal layer adds membrane channels, cargo adaptors, and signalling capacity. Comparisons across fungal lineages indicate substantial variation in flagellar composition, with Neocallimastigomycota retaining the broadest toolkit and showing select expansions amid centriole reductions, pointing to multiple evolutionary trajectories toward motility.

## Methods

Using several sources, we built a comprehensive collection of flagellar proteins by integrating datasets from publicly available databases and literature sources. After removing redundant entries, we performed homology searches in a defined set of model organisms to find more reference proteins. We then combined those proteins into separate protein homology groups and screened them against fungal proteomes. Obtained fungal homologs were then manually cleaned to remove potential false positive hits and narrow-down the results.

Our primary reference dataset comprised proteins from the Complex Portal^51^ (*n* = 188), retrieved using the keyword “cilium,” and from CORUM^52^ (*n* = 210), selected under the predefined categories “cilium assembly” and “cilium movement”. The dataset al.so includes proteins derived from UniProtKB^53^ (*n* = 261), using the keyword “(organism_id:9606) AND (go:0036126)”. In parallel, we incorporated 123 human orthologs of proteins linked to flagellum in *Chlamydomonas reinhardtii*, as reported by Pazour et al. (2005, Supplementary Table S1)^54^ and 87 from Merenyi et al. (2023, Supplementary Data 3)^11^ as well as 62 proteins from the list of centriole associated proteins defined by Long et al. (2025)^13^. Proteins with very broad flagellum-related functions, such as MAP kinases, were not included in the dataset. Redundant entries across these datasets were manually removed and unified, creating a set of 394 homolog groups (Supplementary File S1). The 394 orthogroups were mapped on a set of outgroup taxa using blastp (version 2.13.0+; e-value threshold of 1e-3)^80^. The outgroup set included model animals *Mus musculus* (GCA_000001635.9), *Homo sapiens* (GCA_000001405.29), *Drosophila melanogaster* (GCA_000001215.4), and *Caenorhabditis elegans* (GCA_000002985.3), along with basal *Opisthokonta* representatives: *Monosiga brevicollis* (GCF_000002865.3), *Capsaspora owczarzaki* (GCF_000151315.2), *Salpingoeca rosetta* (GCF_000188695.1), *Fonticula alba* (GCF_000388065.1), and *Sphaeroforma arctica* (GCF_001186125.1), as well as *C. reinhardtii* (GCA_000002595.3). To identify orthologs in orthogroups with many paralogs, we performed phylogenetic analysis (Supplementary Figure S3 - S14). The phylogenetic trees were constructed with IQ-TREE (v1.6.9)^81^ using maximum likelihood method with automated model selection. The trees were visualized and represented using the iTOL online tool^82^.

Homology searches in fungi were performed using 184 fungal proteomes (Supplementary File S1). By proteomes, we define the complete sets of all proteins coded by genes in 184 genome assemblies downloaded from NCBI in May 2022^83^. These comprise all then-available non-Dikarya proteomes and proteomes of representatives of Dikarya classes. To ensure that for each reference protein, sets of reliable fungal homologs were obtained, we checked the conservation of Pfam (database v. 36) protein domains by scanning them with pfamscan.pl v1.6 with its default settings^84^. Each homology group was noted as present or absent from fungi. Additionally, we used tblastn v. 2.13.0 + ^80^ with an e-value threshold of 1e-5 (against 184 fungal genomes) to eliminate potential false negative results resulting from possible incomplete protein sets in proteomes.

Each set of homologsous sequences was subjected to clustering based on amino acid sequence similarity using CLANS (version 2.0)^85^ (with a range of* p*-values of 1e − 10, 1e − 20 and 1e − 30, attraction exponent value of 2 and remaining parameters set to default values). Such a clustering approach allows to remove false positives while retaining the closest homologs for divergent proteins. Protein group categorization has been made based on Long et al. (2025)^13^ and Merényi et al. (2023)^11^ categories as well as on CORUM and ComplexPortal annotations, in addition to UniProtKB^53^ annotations. Main literature sources used to establish each of the functional and structural categories used for the classification of flagellar proteins are listed in Supplementary File S1. Fungal homolog groups were characterized using sequence annotation tools. For the identification of transmembrane helices, TMHMM (version 2.0)^86^ was utilized. Sequence disorder predictions were carried out using IUPred (version 2.0)^87^. SEG (version 1.0)^88^ was employed for the prediction of low-complexity regions. All of the above-mentioned software was used with the default settings. Multiple sequence alignments were constructed via MAFFT (version 7.475)^89^, employing parameters --localpair and --maxiterate 100. Figures were created using Python and Matplotlib^90^.

We performed gene expression analysis taking two publicly available transcriptomic datasets of *Rhizoclosmatium globosum* and its developmental stages^5,58^. The datasets were labelled A and B based on their year of availability in NCBI-SRA (2022 and 2025 respectively). The data were downloaded in the form of fastq files from the ENA server^91^. The RNASeq data analysis was carried out based on the steps described by Pasterny et al., 2025^92^. The average Transcripts Per Million (TPM) values were transformed into log2TPM values. The log2TPM values were used to construct a box plot showing the developmental stages and expression of proteins specific and not specific to flagellated fungi respectively. The box plot was generated using ggplot2^93^. In order to verify the differences in expression levels between flagellated fungi-specific and non-specific proteins, we used the Mann-Whitney U test/ Wilcoxon Rank sum test in R^94,95^. This non-parametric approach tested the null hypothesis that both groups share the same distribution, with the obtained p-values as an indicator of significance.

## Supplementary Information

Below is the link to the electronic supplementary material.

Supplementary Material 1

Supplementary Material 2

## Data Availability

All metadata processed in this study are deposited in zenodo: https://doi.org/10.5281/zenodo.19707388All protein identifiers and genomic assemblies are listed in Supplementary file S1.
