# Supplementary material for: A conserved minimal core and modular extensions make the fungal flagellum
Source: Sci Rep. 2026 May 12;16:21656. doi: 10.1038/s41598-026-52644-y (PMC13358043; doi:10.1038/s41598-026-52644-y)
Supplement: Supplementary file 2 — Supplementary Material 2 [file 41598_2026_52644_MOESM2_ESM.pdf]

A conserved minimal core and modular extensions make the fungal flagellum

Aleksander Kossakowski[1,2] and Anna Muszewska[1,*]

[1] Institute of Biochemistry and Biophysics, Polish Academy of Sciences, Pawińskiego 5A, 02-106 Warsaw, Poland

[2] Doctoral School of Molecular Biology and Biological Chemistry at IBB PAS, Pawinskiego 5a, 02-106 Warsaw, Poland

* corresponding author: Anna Muszewska musze@ibb.waw.pl

LIST OF SUPPLEMENTARY FIGURES

Supplementary Figure S1 - Taxonomic distribution of flagellum orthogroups................2
Supplementary Figure S2 - Consecutive duplications of DAAM1/2 paralogs in Neocallimastigomycota.. 3
Supplementary Figure S3 - Phylogenetic tree of RIBC1/2.. 4
Supplementary Figure S4 - Phylogenetic tree of RSPH4/6... 5
Supplementary Figure S5 - Phylogenetic tree of TSSK4/6..6
Supplementary Figure S6 - Phylogenetic tree of CALM1/2/3..7
Supplementary Figure S7 - Phylogenetic tree of CETN1/2/3.. 8
Supplementary Figure S8 - Phylogenetic tree of SEPTINS...9
Supplementary Figure S9 - Phylogenetic tree of KIF3A, KIF3B/C/17.............................10
Supplementary Figure S10 - Phylogenetic tree of DRC1, DRC2......................................11
Supplementary Figure S11 - Phylogenetic tree of TTLL3/8...12
Supplementary Figure S12 - Phylogenetic tree of DYNLT1/3 and DYNLT2/4................. 13
Supplementary Figure S13 - Phylogenetic tree of DYNLL1/2... 14
Supplementary Figure S14 - Phylogenetic tree of dynein heavy chains........................ 15

Fungal sequences are denoted in green, reference sequences in pink. The phylogenetic trees were constructed with IQ-TREE (v1.6.9) using maximum likelihood method with automated model selection and ultrafast bootstrap. The trees were visualized and represented using the iTOL online tool.

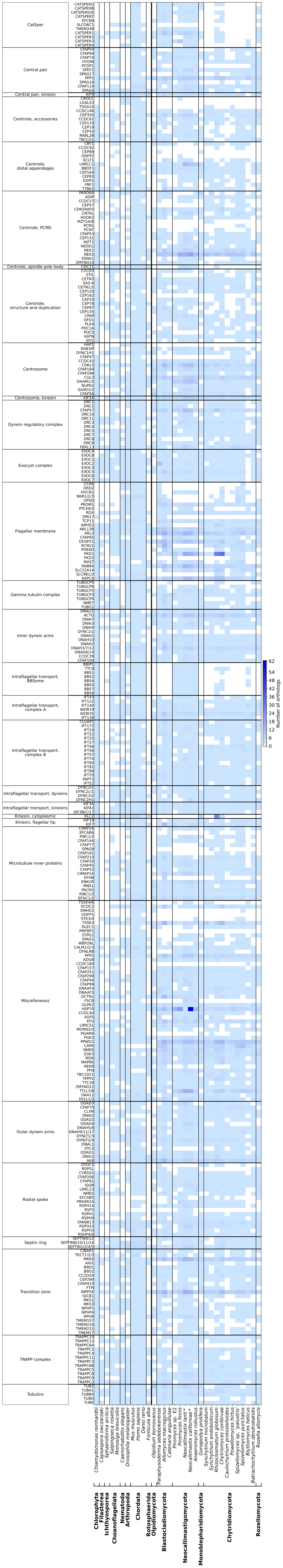
CatSper
Central pair
Central pair, kinesin
Centriole, accessories
Centriole, distal appendages
Centriole, PCMS
Centriole, spindle pole body
Centriole, structure and duplication
Centrosome
Centrosome, kinesin
Dynein regulatory complex
Exocyst complex
Flagellar membrane
Gamma tubulin complex
Inner dynein arms
Intraflagellar transport, BBSome
Intraflagellar transport, complex A
Intraflagellar transport, complex B
Intraflagellar transport, dyneins
Intraflagellar transport, kinesins
Kinesin, cytoplasmic
Kinesin, flagellar tip
Microtubule inner proteins
Miscellaneous
Outer dynein arms
Radial spoke
Septin ring
Transition zone
TRAPP complex
Tubulins
Number of orthologs
0
6
12
18
24
30
36
42
48
54
62
Chlorophyta
Filasterea
Ichthyosporea
Choanoflagellata
Nematoda
Arthropoda
Chordata
Rotosphaerida
Olpidiomycota
Blastocladiomycota
Neocallimastigomycota
Monoblepharidiomycota
Chytridiomycota
Rozellomycota

Supplementary Figure S1 - Taxonomic distribution of flagellum orthogroups.

This heatmap shows the presence and abundance of 342 flagellum-related orthogroups present in reference organisms and flagellated fungi.

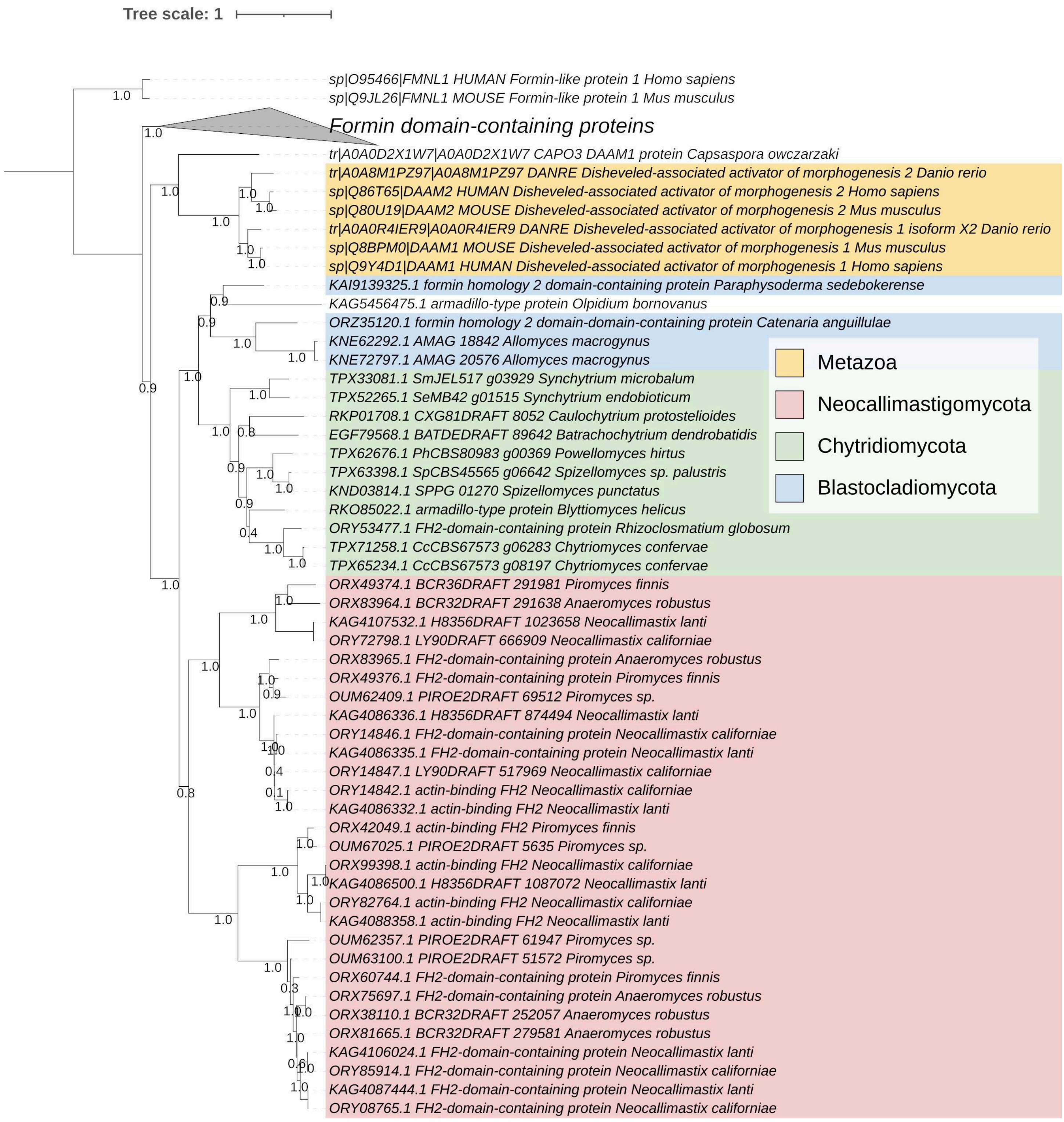

Supplementary Figure S2 - Consecutive duplications of DAAM1/2 paralogs in Neocallimastigomycota.

Phylogenetic tree of chosen 80 formin-like proteins in reference organisms and flagellated fungi, showing expansion of DAAM1/2 proteins in Neocallimastigomycota.

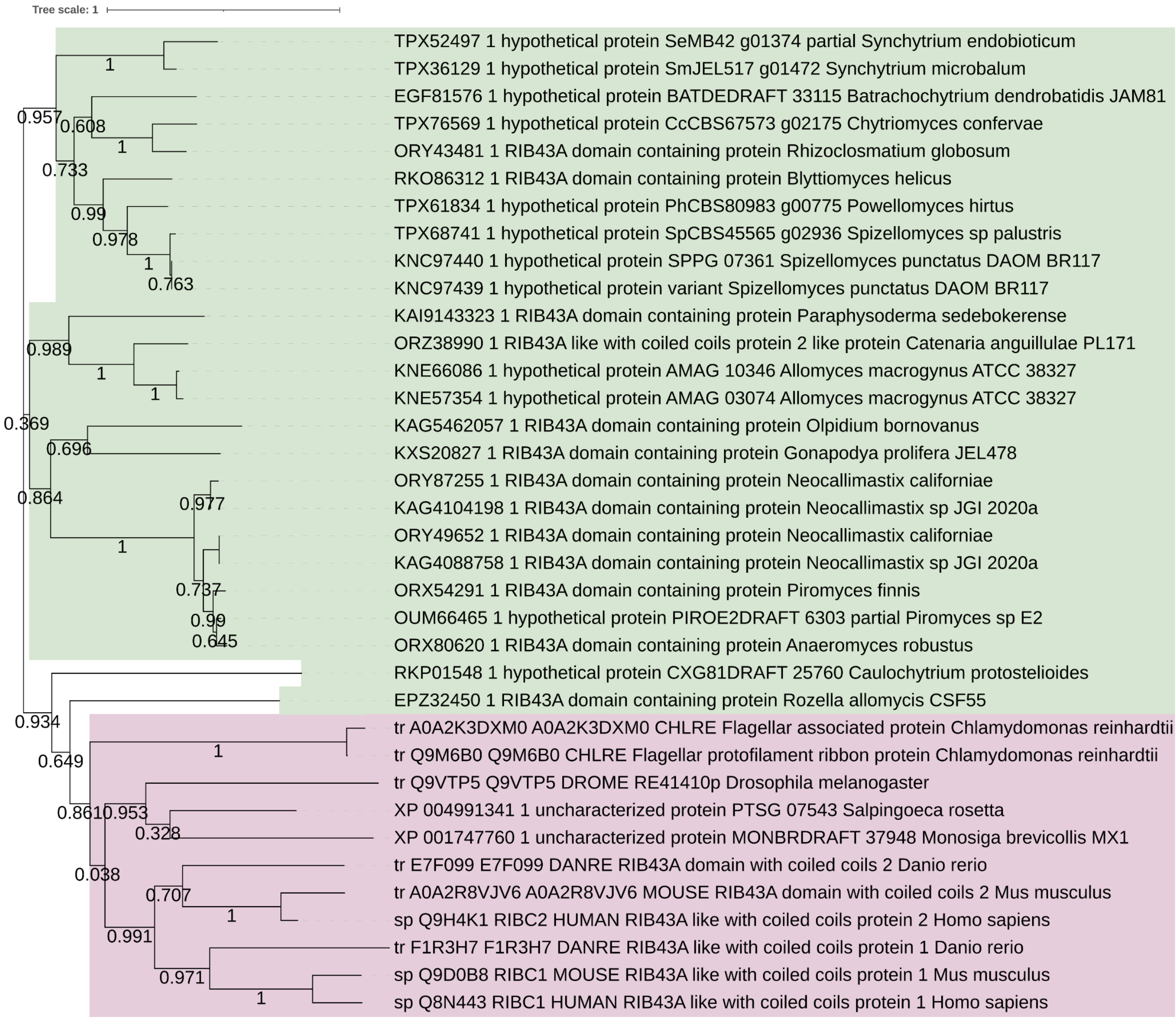

Supplementary Figure S3 - Phylogenetic tree of RIBC1/2

Phylogenetic tree of 37 sequences of RIBC1/2 and their fungal homologs showing a single monophyletic clade for reference sequences suggesting the duplication of RIBC1 in Vertebrata.

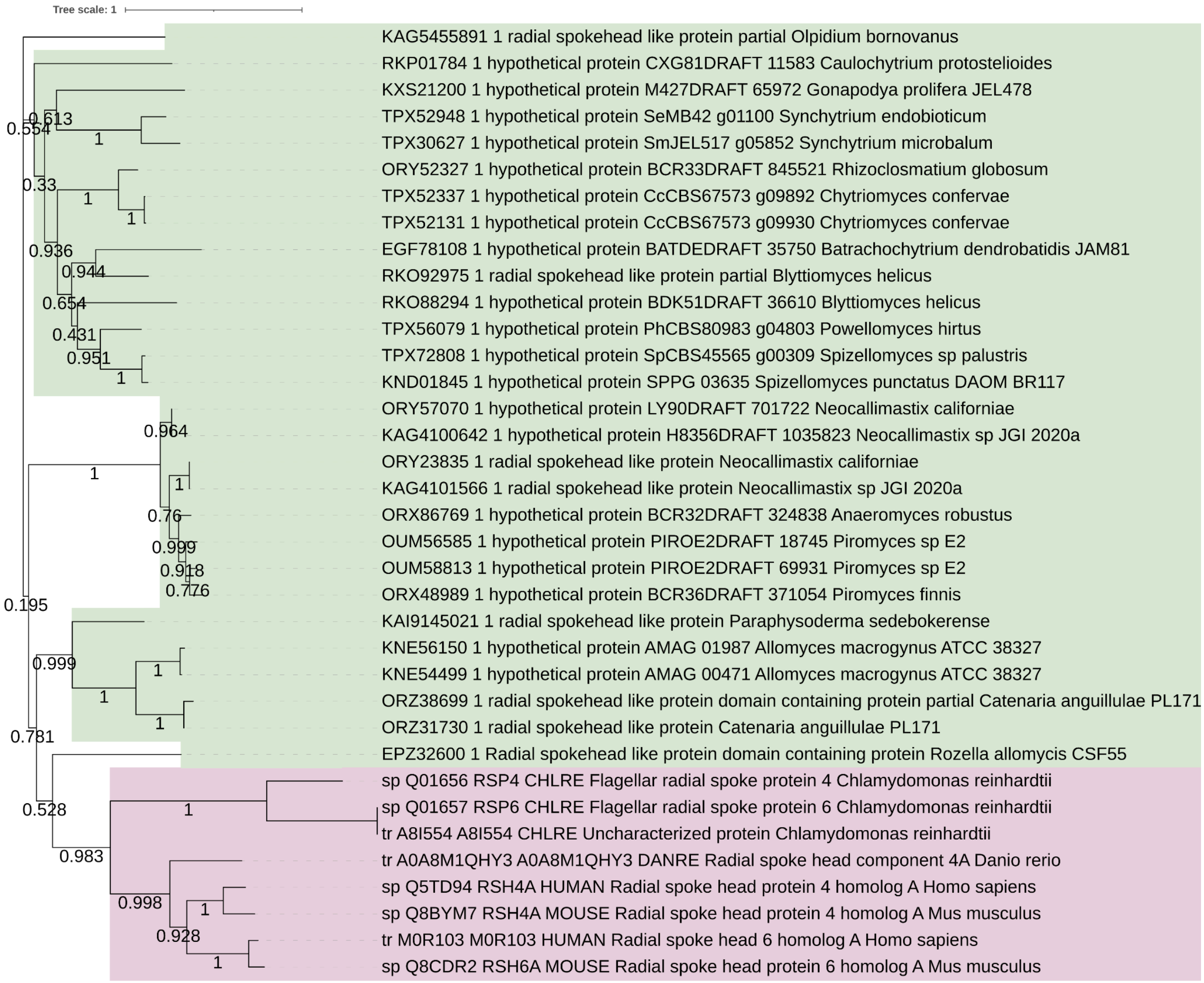

Supplementary Figure S4 - Phylogenetic tree of RSPH4/6

Phylogenetic tree of 29 sequences of RSPH4/6 and their fungal homologs that suggests the presence of one RSPH4/6 copy in most of the analysed fungi.

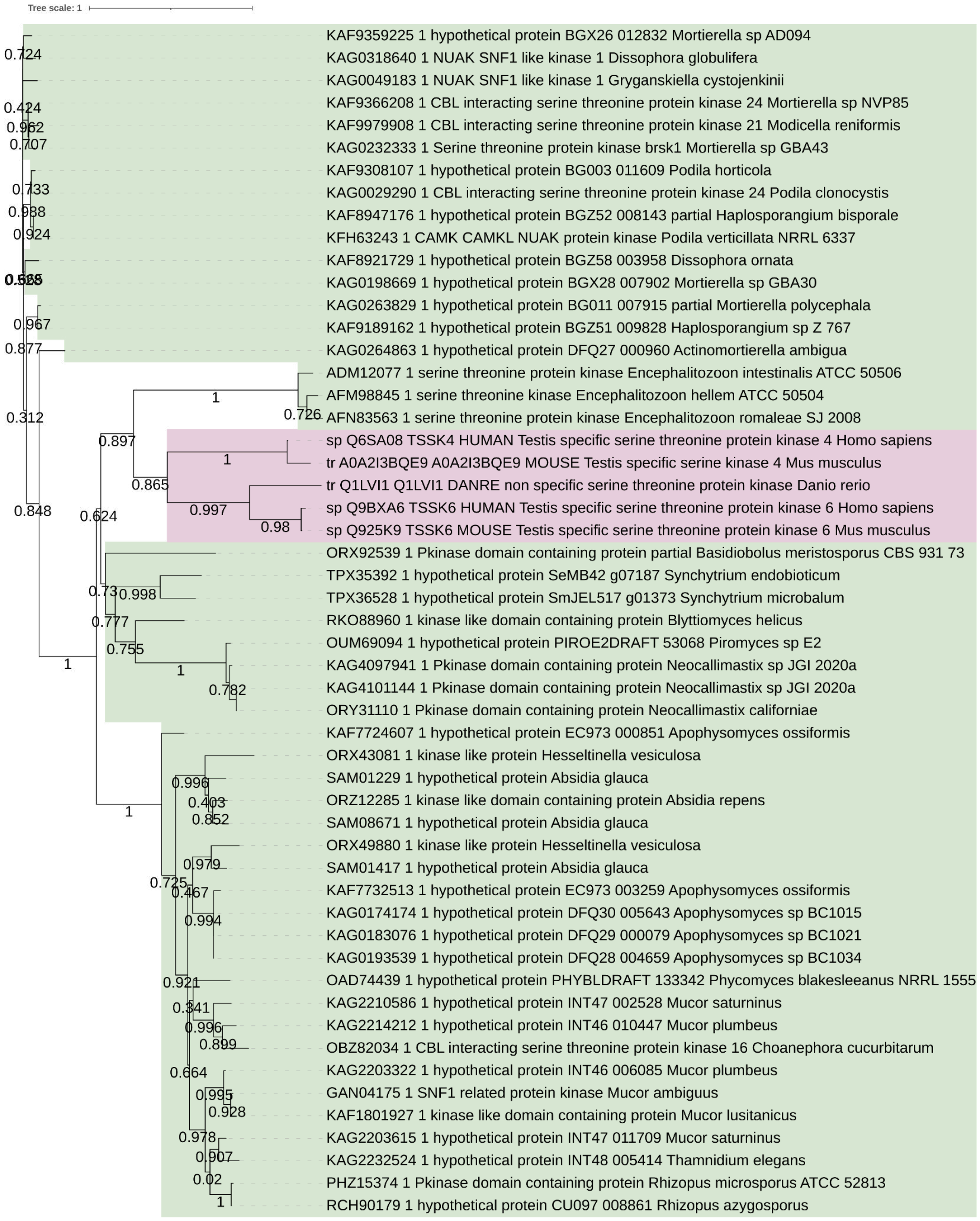

Supplementary Figure S5 - Phylogenetic tree of TSSK4/6

Phylogenetic tree of 49 sequences of TSSK4/6 and their fungal homologs that recapitulates the fungal species tree including nonflagellated representatives of Mucoromycota and Mortierellomycota.

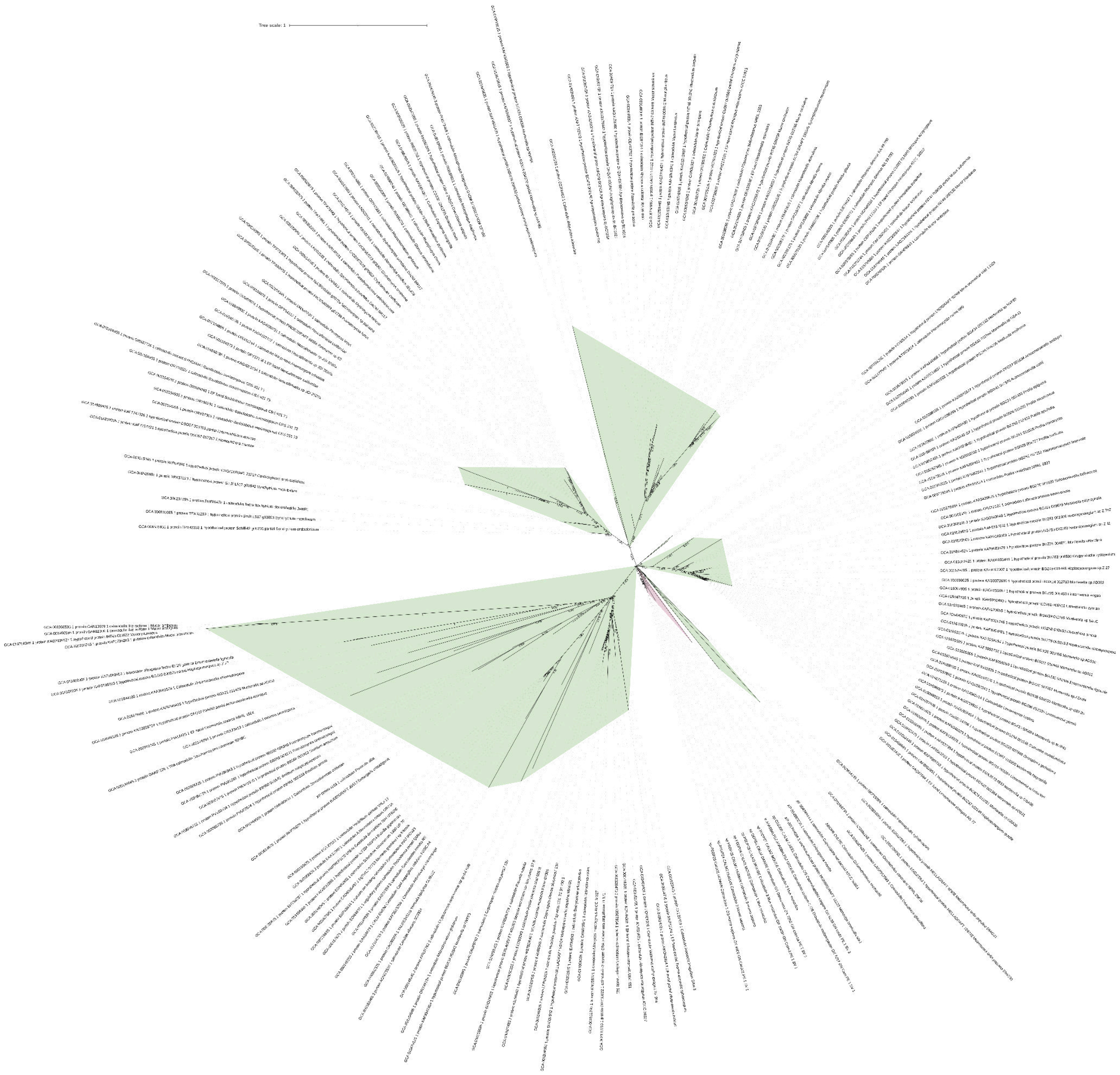

Supplementary Figure S6 - Phylogenetic tree of CALM1/2/3

Phylogenetic tree of 182 sequences of CALM1/2/3 and their fungal homologs. All three human CALM1/2/3 and three mouse CALM1/2/3 calmodulin paralogs are identical at the amino acid sequence levels, and in consequence they all form a monophyletic clade.

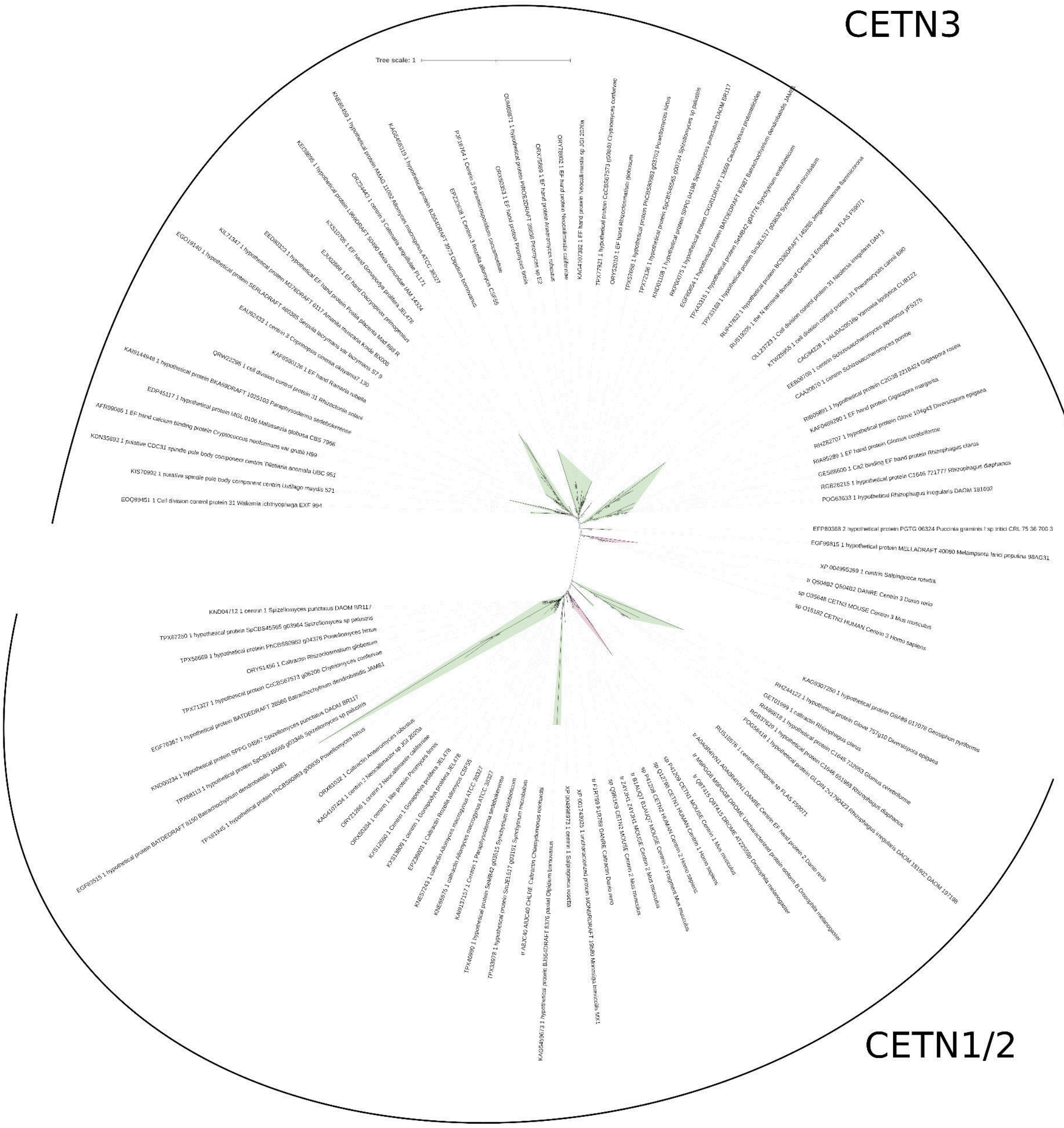

Supplementary Figure S7 - Phylogenetic tree of CETN1/2/3

Phylogenetic tree of 84 sequences of CETN1/2/3 and their fungal homologs shows two separated clades, one grouping CETN1&2 and the latter only CETN3 homologs.

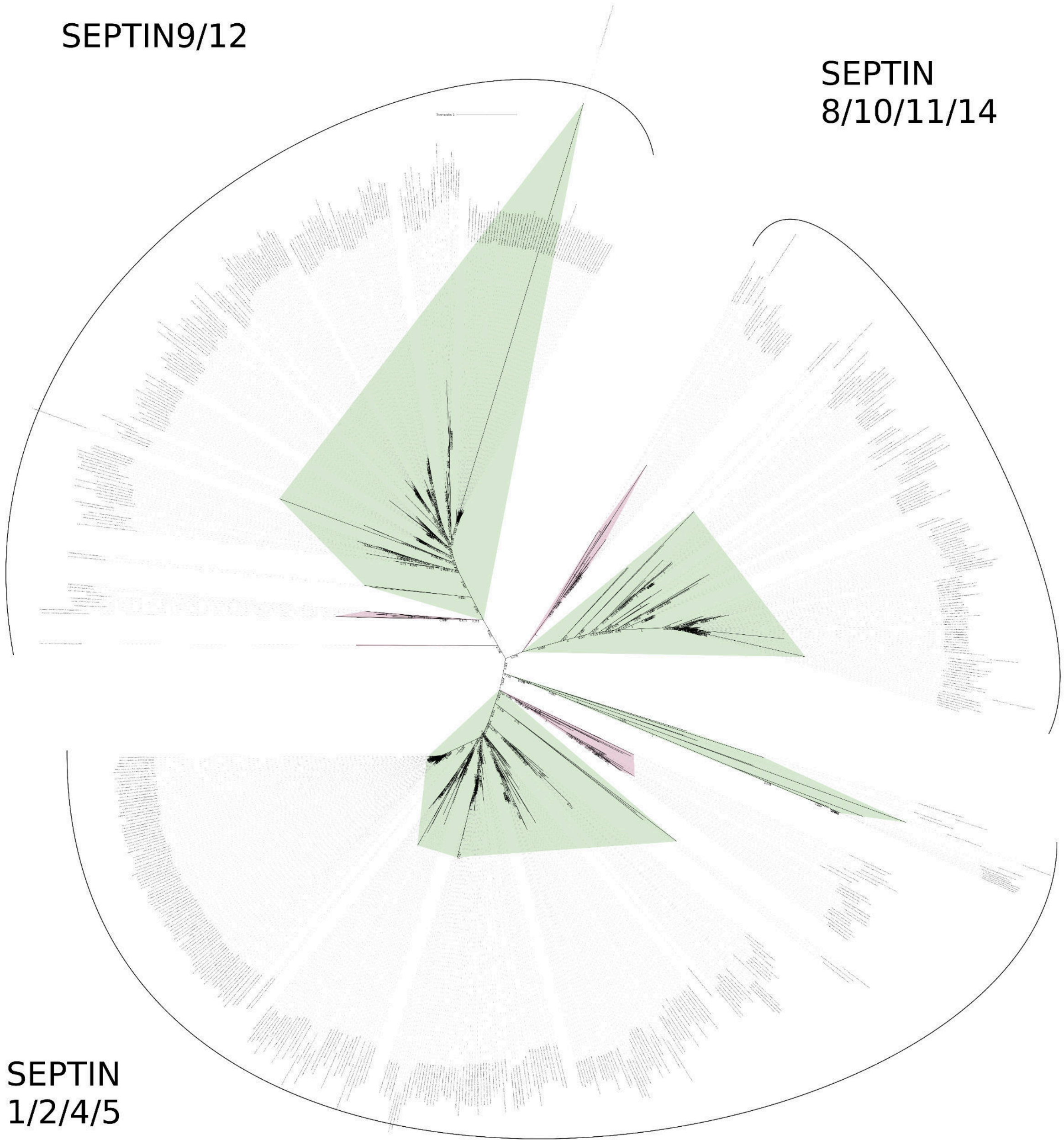

Supplementary Figure S8 - Phylogenetic tree of SEPTINS

Phylogenetic tree of 652 sequences of SEPTINS and their fungal homologs showing that SEPTIN1/2/4/5, SEPTIN9/12 and SEPTIN8/10/11/14 form separate clades.

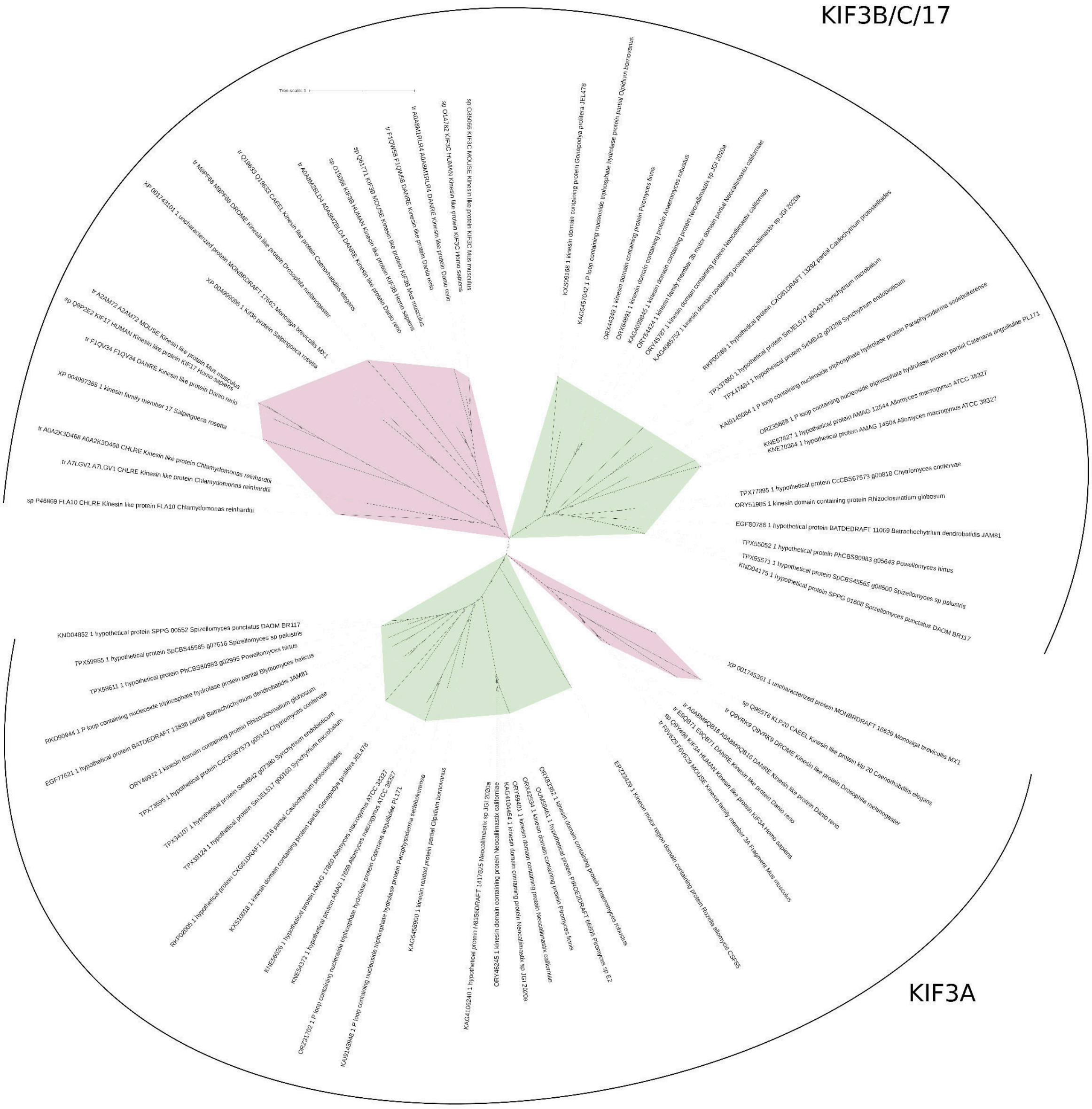

Supplementary Figure S9 - Phylogenetic tree of KIF3A, KIF3B/C/17

Phylogenetic tree of 49 sequences of KIF3A, KIF3B/C/17 and their fungal homologs that form two main clades one with KIF3A and a second with KIF3B/C/17 homologs.

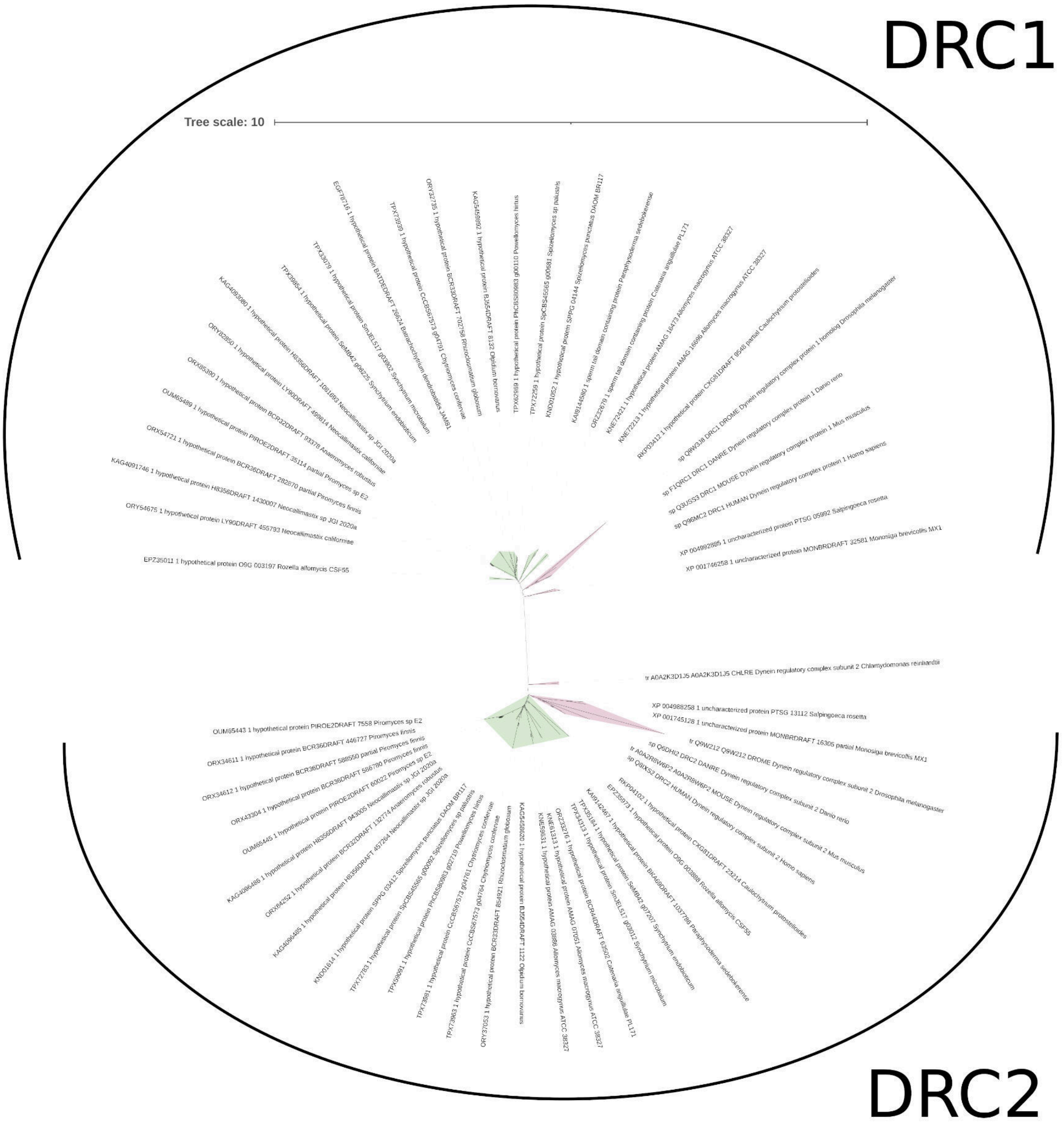

Supplementary Figure S10 - Phylogenetic tree of DRC1, DRC2

Phylogenetic tree of 49 sequences of DRC1, DRC2 and their fungal homologs which shows two separate clades, one with DRC1 and other with DRC2.

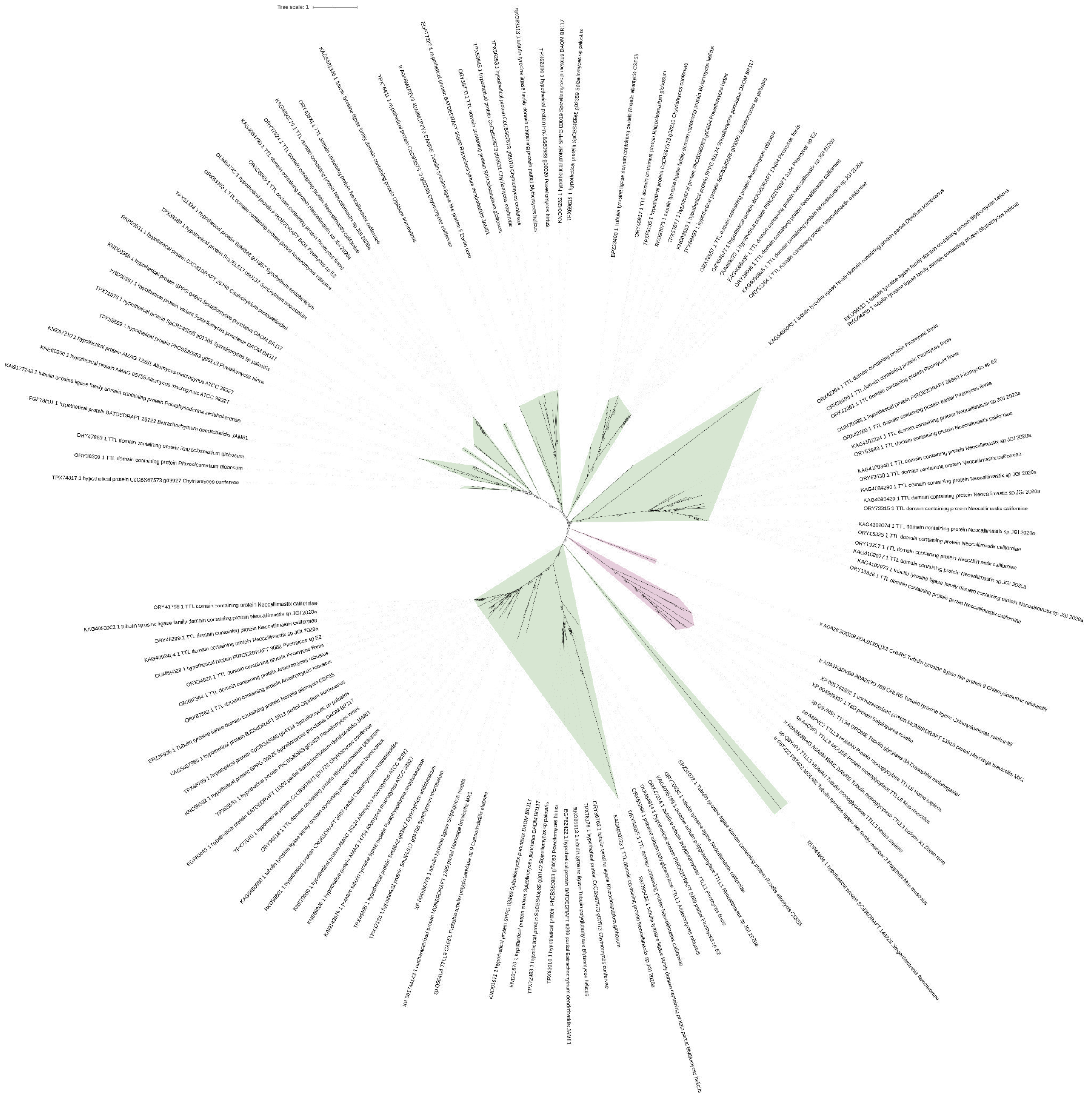

Supplementary Figure S11 - Phylogenetic tree of TTLL3/8

Phylogenetic tree of 112 sequences of TTLL3/8 and their fungal homologs showing one clade with TTLL3 and TTLL8.

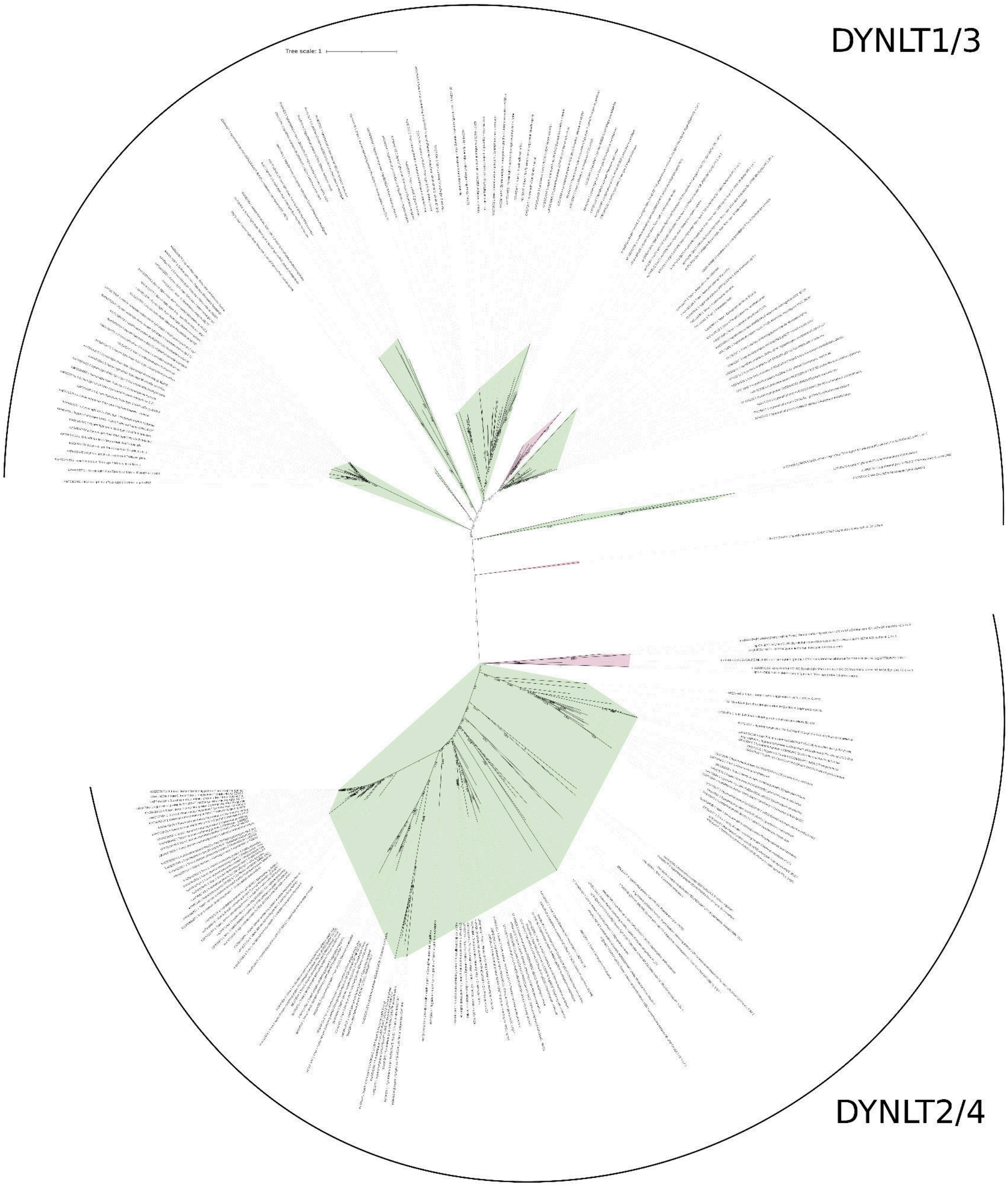

Supplementary Figure S12 - Phylogenetic tree of DYNLT1/3 and DYNLT2/4

Phylogenetic tree of 232 sequences of DYNLT1/3 and DYNLT2/4 and their fungal homologs showing two separate clades, one formed by DYNLT1/3 and other by DYNLT2/4.

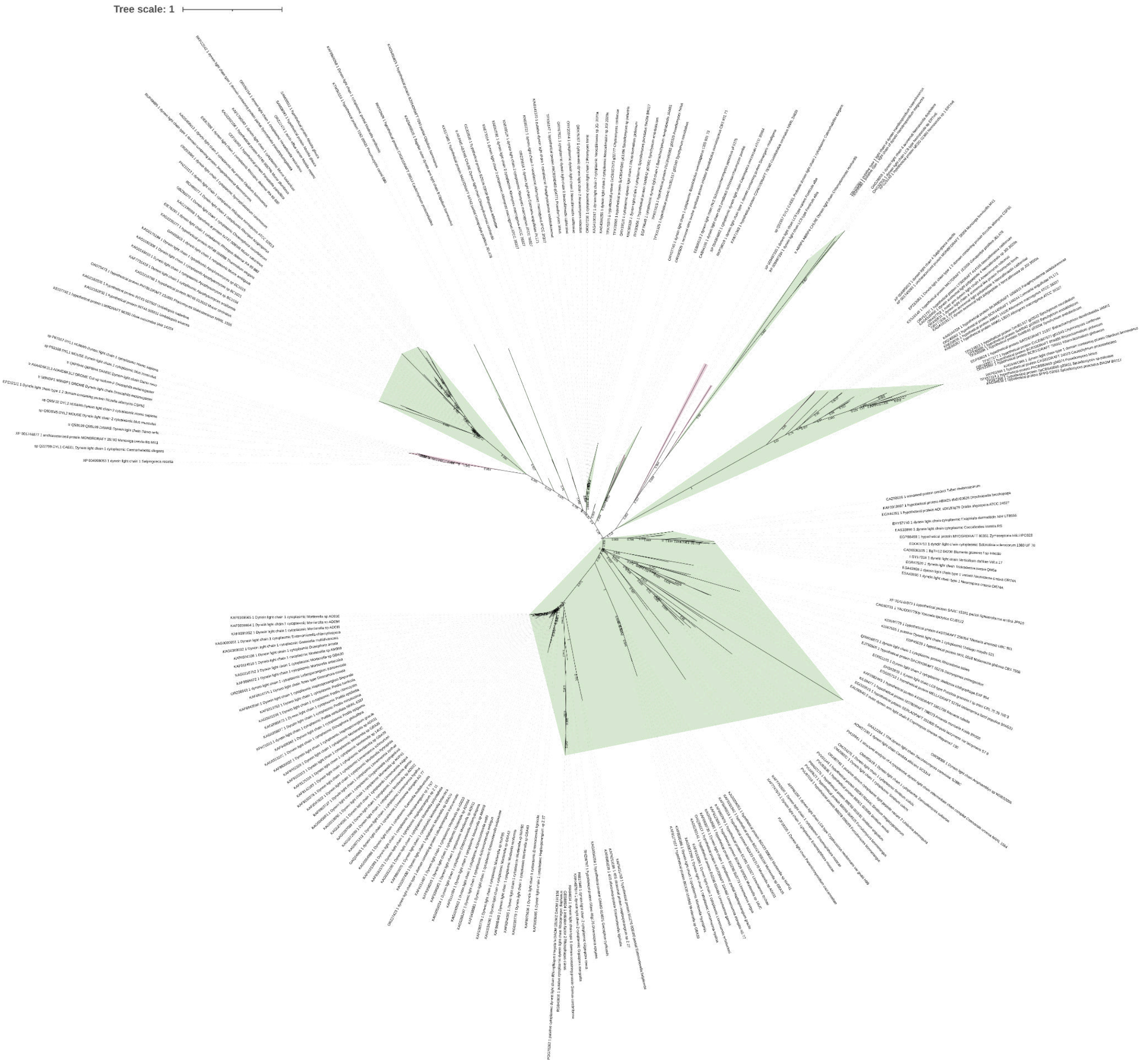

Supplementary Figure S13 - Phylogenetic tree of DYNLL1/2

Phylogenetic tree of 234 sequences of DYNLL1/2 and their fungal homologs showing that human and mouse DYNLL1 and DYNLL2 form one clade within the tree.

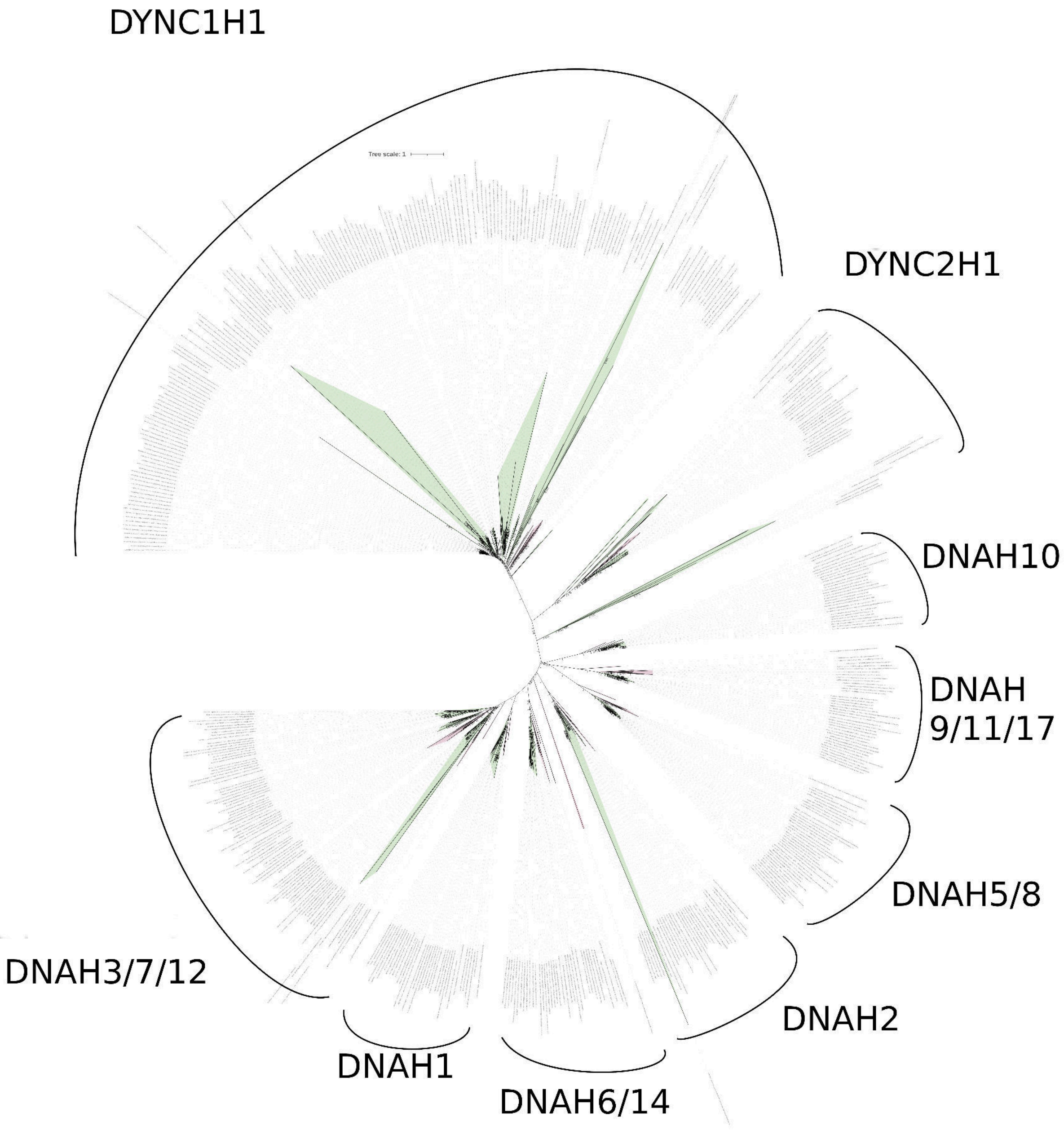

Supplementary Figure S14 - Phylogenetic tree of dynein heavy chains

Phylogenetic tree of 540 sequences of DNAH and DYNC1H1, DYNC2H1 families and their fungal homologs showing different groups of flagellated fungi specific DNAHs, and more ubiquitously occurring DYNC1H1 and DYNC2H1.
